# Evaluating Caveolin Interactions: Do Proteins Interact with the Caveolin Scaffolding Domain through a Widespread Aromatic Residue-Rich Motif?

**DOI:** 10.1371/journal.pone.0044879

**Published:** 2012-09-17

**Authors:** Dominic P. Byrne, Caroline Dart, Daniel J. Rigden

**Affiliations:** Institute of Integrative Biology, The University of Liverpool, Liverpool, United Kingdom; University College Dublin, Ireland

## Abstract

Caveolins are coat proteins of caveolae, small flask-shaped pits of the plasma membranes of most cells. Aside from roles in caveolae formation, caveolins recruit, retain and regulate many caveolae-associated signalling molecules. Caveolin-protein interactions are commonly considered to occur between a ∼20 amino acid region within caveolin, the caveolin scaffolding domain (CSD), and an aromatic-rich caveolin binding motif (CBM) on the binding partner (фXфXXXXф, фXXXXфXXф or фXфXXXXфXXф, where ф is an aromatic and X an unspecified amino acid). The CBM resembles a typical linear motif - a short, simple sequence independently evolved many times in different proteins for a specific function. Here we exploit recent improvements in bioinformatics tools and in our understanding of linear motifs to critically examine the role of CBMs in caveolin interactions. We find that sequences conforming to the CBM occur in 30% of human proteins, but find no evidence for their statistical enrichment in the caveolin interactome. Furthermore, sequence- and structure-based considerations suggest that CBMs do not have characteristics commonly associated with true interaction motifs. Analysis of the relative solvent accessible area of putative CBMs shows that the majority of their aromatic residues are buried within the protein and are thus unlikely to interact directly with caveolin, but may instead be important for protein structural stability. Together, these findings suggest that the canonical CBM may not be a common characteristic of caveolin-target interactions and that interfaces between caveolin and targets may be more structurally diverse than presently appreciated.

## Introduction

Caveolins are a family of cholesterol-binding membrane proteins (caveolin-1, -2 and -3) that coat the intracellular surface of caveolae, small flask-shaped pits (50–100 nm in diameter) that form at the plasma membrane of most cells [1]–[4]. Aside from roles in caveolae formation and stability, caveolins interact with many caveolae-localized signalling molecules including heterotrimeric G proteins, Src family tyrosine kinases, phosphoinositide 3-kinase, integrins, epidermal growth factor receptor (EGFR), H-Ras, endothelial nitric oxide synthase (eNOS) and a number of ion channels [3], [5]. Interaction with caveolin, which appears to be important in protein recruitment to caveolar domains and thus the formation of microenvironments rich in interacting signalling molecules, is commonly believed to be mediated via a ∼20 amino acid N-terminal region on the caveolin molecule known as the caveolin scaffolding domain (CSD) and an aromatic-rich caveolin binding motif (CBM) on the associated protein [6], [7]. Paradoxically, association with caveolin typically suppresses activity in the targeted protein [6], [7], suggesting that recruitment to caveolae might hamper and not enhance signalling efficiency (the so-called ‘caveolar paradox’). This paradox has been largely resolved for eNOS whereby interaction with caveolin under basal conditions maintains an inactive enzyme and compartmentalization of eNOS in caveolae ensures a rapid response upon stimulation [8]. Interactions between caveolin and other proteins, however, remain poorly understood in terms of physiology, modes of binding/suppression and the mechanisms that regulate interaction.

Since the original definitions of the CSD and CBM, an increasing number of studies have suggested that interactions between caveolin and target need not necessarily involve both regions. Association of caveolin with NOSTRIN [9], cyclooxygenase-2 [10], high affinity nerve growth factor receptor (Trk [11]), growth factor receptor-bound protein 7 (Grb7 [12]) and insulin receptor substrate 1 (IRS1 [13]) are all thought to occur independently of the CSD. Furthermore, in some cases interactions appear to occur via multiple distinct caveolin domains. For example, interaction with protein kinase A is dependent on either the CSD or C-terminal domain (amino acids 135–178) of Cav-1 [14]. Dynamin-2, endothelin-B, connexin-43 and Rab5 also interact with multiple distinct regions of Cav-1 [15]–[18]. Target association with the caveolin scaffolding domain is mainly proposed to occur via the caveolin binding motif (CBM) on the binding partner. The original definition of the CBM arises from the work of Couet *et al*., who obtained random peptides binding to the CSD by phage display [6]. The peptides obtained were statistically enriched in tryptophan (decapeptides and 15-mers) or other aromatic amino acids (15-mers). Noting that certain separations of aromatic residues were particularly common, the authors identified a 16-residue portion of the bovine Gi2α subunit (the GP peptide) which bound to CSDs from caveolin-1 and 3 and much less so to caveolin 2. When all four aromatic residues were simultaneously mutated to Ala or Gly the interaction was lost. Based on this finding three CBM variants were defined, each containing three or four aromatic residues separated by unspecified amino-acids (CBMs: **Ф**X**Ф**XXXX**Ф**, **Ф**XXXX**Ф**XX**Ф** or **Ф**X**Ф**XXXX**Ф**XX**Ф**, where **Ф** is an aromatic amino acid), and shown to occur in known or possible caveolin binding proteins. Although the notion of these aromatic-rich motifs has figured prominently in the literature, the fact that the four aromatic positions in the caveolin binding peptide were not independently mutated means that there is no reason to suppose that all four should invariably be present in CBM sequences. Equally, the quadruple mutation would be expected to have dramatic effects on any tertiary structure that the GP peptide might have, raising doubts as to whether the aromatic residues function in direct binding or have an indirect role in stabilising the active peptide conformation.

There are several cases where binding to caveolin occurs entirely independently of a typical CBM. For example, Sprouty-protein 1, which lacks a CBM, binds Cav-1 via its conserved cysteine-rich C-terminal domain, an interaction which is completely eliminated by a single amino acid exchange mutation (R252D [19]). Hepatocyte cell adhesion molecule (hepaCAM), binds Cav-1 via the first immunoglobulin domain which also lacks a traditional CBM domain [20]. Binding of Cav-1 to DNA-binding protein inhibitor, ID-1, occurs via a helix-loop-helix domain, a region lacking a typical CBM [21]. The catalytic domain of protein kinase A (PKAcat), nerve growth factor receptor, and sterol carrier protein also bind Cav-1, despite lacking CBM sequences [21]. Furthermore, there are also cases of proteins containing CBMs that do not bind to caveolin: both RhoA and RhoB have identical CBM sequences, yet only the former localises with Cav-1 in caveolae [22]. Likewise, an 'incomplete CBM' is also found in low molecular weight protein tyrosine phosphatase (^78^ ITKED**F**AT**F** ^86^) but is not recognised as the binding site for Cav-1 [23]. Together, these findings suggest that the CBM, like the CSD (see above) is not necessarily required for all caveolin interactions. At this point it should be noted that, although many caveolin binding proteins have been described, in many cases it is unclear if these are direct interactions or whether they are facilitated indirectly via intermediary molecules of a larger caveolin-containing complex. Thus, it is possible that regions predicted to be crucial for caveolin interaction (including sequences resembling CBMs) may function by binding intermediary molecules which then recruit caveolin.

The CBM, as proposed, is a prime example of a short, linear motif (SLiM) - a simple sequence that would have independently evolved many times in different proteins for a specific function, in this case binding to the CSD. Until recently the fundamental role of such motifs in mediating the protein-protein interactions underlying cellular regulation and signalling has been under-appreciated. Such SLiMs have presented significant bioinformatics challenges. However, recent years have seen major advances in detection of interaction motifs through their over-representation in interactome sequences [24]–[26], benefiting especially from knowledge that SLiMs tend to be conserved and positioned preferentially in intrinsically disordered parts of proteins [26]. Other recently developed methods use these criteria and others, such as predicted solvent exposure and secondary structure [27] or energetic factors [28], to predict potential motifs in single sequences. Weatheritt *et al*. [29] have also described a method to identify SLiM interaction interfaces for both interacting proteins. Here we exploit these recent improvements in bioinformatics techniques available for the study of linear motifs to critically examine the role of aromatic-rich CBMs in caveolin interactions. We assess their frequency of occurrence in the human proteome, their statistical enrichment in the caveolin interactome and shared characteristics with other known interaction motifs. We examine the relative solvent accessible area (RSA) of the CBM aromatic residues for Cav-1 interaction partners in solved crystal structures and homology models to assess the likelihood that the conserved aromatics are available for direct binding of proteins. Finally, we calculate the predicted ΔΔG free energy stability change resulting from point mutations of the aromatic residues to examine their role in protein stability. Our findings suggest that the CBM, despite its prevalence in the caveolin literature, is not required for all caveolin interactions and may in fact only be genuinely implicated in a small minority of cases. This conclusion is significant for future caveolin research.

## Results

### Experimental Evidence Regarding CBMs as Mediators of Caveolin Interaction

Aromatic-rich putative CBMs have been identified in numerous caveolin associated molecules (Table 1). In some work large aliphatic residues such as Leu are considered as substitutes for the aromatic positions (Table 2). For a few proteins there is some supporting evidence demonstrating that the putative CBM mediates interaction with the CSD (i.e. targeted mutation of the CBM disrupts caveolin binding). For example, deletion of the entire CBM (^1130^ **Y**NMLC**F**GI**Y** ^1138^) of the large conductance, voltage- and Ca^2+^-activated potassium channel α-subunit (Slo1) causes ∼80–85% loss of Slo1-Cav-1 association [30]. Some authors have also reported active roles for the individual aromatic residues of CBMs. For example, simultaneous mutation of all three aromatics (^376^ **W**S**F**AVLL**W** ^383^) in the integrin-linked protein kinase abolishes Cav-1 binding [31]. Two serine/threonine-protein kinase receptor R3 CBM mutants (W406A; F401G and W406A) also exhibit substantial reduction in co-immunoprecipitation with Cav-1 [32]. Kong *et al*. [33] created several D(1A) dopamine receptor mutants with disrupted proximal, central and distal CBM aromatic residues which exhibited reduced binding affinity for caveolin. Point mutation of just one or all three CBM aromatics of ephrin type-B receptor 1 (EphB1) receptor also severely reduced receptor co-immunoprecipitation with Cav-1 [34]. Glucagon-like peptide 1 receptor also fails to interact with caveolin following mutation of two tyrosine residues within the motif [35]. Site-directed mutagenesis of metabolic glutamate receptor, 3-phosphoinositide-dependent protein kinase 1 (PDK1), phosphatidylinositol-3,4,5-trisphosphate 3-phosphatase, dual-specificity protein phosphatase (PTEN), and sialidase also suggests that interaction with Cav-1 is mediated by the CBM [36]–[39]. Similarly, haem-oxygenase-1 possesses an incomplete CBM motif (^227^ **F**LLNIQL**F** ^234^) and completely loses affinity for the Cav-1 CSD following mutation of the motif’s two Phe residues (F227 and F234 [40]). However, in the main, there seems to be little unambiguous evidence that these motifs, and crucially the positioning of their aromatic amino acid residues, are generally required for caveolin interactions. Several examples were mentioned in the introduction of proteins in which caveolin interaction has proved to be independent of any CBM-like sequence. In other examples, mutagenesis of putative CBMs fails to show a substantial effect on caveolin interaction. For example, a W1227T mutant that disrupts the CBM of the insulin receptor (^1220^ **W**S**F**GVVL**W** ^1227^) still exhibits significant interaction with Cav-1 [41]. Moreover, simultaneous mutation of the CBM aromatic residues Y42A and W45A of the multidrug resistance protein-1 (MDR1) only diminishes interaction with Cav-1 by 27% [42]. It seems highly unlikely that the MDR1 CBM could still function as such a potent interface for Cav-1 binding while possessing just one remaining functional motif residue, which strongly implicates non-CBM residues as the mediators of Cav-1 binding. Furthermore individual F589L and W592L mutations of the neuronal nitric oxide synthase (nNOS) CBM resulted in only slight reductions of the Cav-1 inhibitory effect (IC50 values of 3.5 and 3.0 µM respectively compared to 1.8 µM for the wild-type protein) suggesting that the motif is also not essential for Cav-1 binding to nNOS [43]. Similarly, despite deletion of the Slo1 CBM greatly reducing Cav-1 interaction, individual point mutation of the aromatics within the motif has a less obvious effect on binding [30]. Whereas F1135A or Y1138A mutations decrease Cav-1-Slo1 association by only ∼15% each, Y1130A increases the interaction by ∼40%. Furthermore, a triple mutation, where all aromatics were mutated, had practically no impact on Cav-1-Slo1 association, suggesting that the mutations had an additive effect and also indicating that other residues within or around the motif stabilize the interaction [30]. The idea that neighbouring residues can also be important is supported by Syme *et al*. [35] who demonstrated that interaction between Cav-1 and the glucagon-like peptide 1 receptor was inhibited by mutation of two aromatic within the proposed CBM (Y250/252A) but also by mutation of a nearby glutamate residue (E247A) outside of the CBM. However, Brainard *et al*. [44] have published contradictory evidence to Alioua *et al.* [30] demonstrating that mutation of all three of the Slo1 CBM aromatics is sufficient to completely abolish Cav-1 interaction.

**Table 1:** List of Cav-1 interacting molecules reported as containing a CBM.

Caveolin associated molecule	CBM sequences and location(aromatic positions emboldened)	Experimental mutation of CBM	Confirmation of structural integrity of mutant	References
ABPP	757-**Y**ENPT**Y**KF**F**-764	No	−	[73]
Adenosine receptor A1	288-**Y**A**F**RIQK**F-**295	No	−	[74]
Aquaporin 1	210-**W**I**F**WVGP**F**-217	No	−	[75]
Beta-adrenergic receptor kinase 1	576-**W**QRRY**F**YQ**F**-584	No	−	[76]
Btk	581-**W**A**F**GVLM**W**-588	No	−	[77]
cGMP-inhibited 3',5'-cyclic phosphodiesterase B	47-**F**F**F**HLCR**F**-54330-**W**D**W**DLKQ**W**-337	No	−	[78]
Cytosolic phospholipase A2	683-**F**Q**Y**PNQA**F**-690	No	−	[79]
D(1A) Dopamine receptor	313-**F**DVFV**W**FG**W**-321	Yes	No	[33]
EGFR	898-**W**S**Y**GVTV**W**-905	No	−	[80]
Ephrin type-B receptor 1	808-**W**S**Y**GIVM**W**-815	Yes	No	[34]
Fatty acid synthase	1506-**Y**RDGA**W**GA**F**-1514	No	−	[81]
Fibroblast growth factor receptor 1	684-**W**S**F**GVLL**W**EI**F**-694	No	−	[82]
Gi2 subunit-α	190-**F**T**F**KDLH**F**KM**F**-200	No	−	[6]
Glucagon-like peptide 1 receptor	247-EGV**Y**L**Y**TLLA**F**-257	Yes	No	[35]
Inositol 1,4,5-triphosphate receptor type 1	218-**W**KIVL**F**MK**W**-226	No	−	[83]
	2461-**Y**L**F**SIVG**Y**-2468	No	−	[83]
Inositol 1,4,5-trisphosphate receptor type 3	219-**W**KINL**F**MQ**F**-227729-**Y**R**Y**QLKL**F**-7362381-**Y**L**F**SIVG**F**L**F**LKDD**F**-2395	No	−	[84]
Insulin receptor	1220-**W**S**F**GVVL**W**-1227	Yes	No	[41], [85]
Integrin-linked protein kinase	376-**W**S**F**AVLL**W**-383	Yes	No	[31]
IBP-3	261-**F**C**W**CVDK**Y**-268	No	−	[86]
Interleukin-6 receptor subunit beta	606-**F**T**F**TTPK**F**-613	No	−	[87]
Kv1.3	216-**F**QRQV**W**LL**F-**224	No	−	[88]
Kv1.5	232-**F**QRQV**W**LI**F**-240	No	−	[88]
Leukemia inhibitory factor receptor	323-**F**GTVV**F**AG**Y**-331	No	−	[87]
MAL-like protein	143-**Y**ILHA**F**SI**Y**-151	No	−	[89]
	23-**F**LTIP**F**AF**F**-31	No	−	[89]
Metabolic glutamate receptor 1	609-**F**VTLI**F**VL**Y**-617	Yes	No	[38]
	781-**F**NEAK**Y**IA**F**-789	Yes	No	
Metalloreductase STEAP4	192-**Y**PLQL**F**PM**W**-200	No	−	[90]
	271-**Y**RGTK**Y**RR**F**-279	No	−	[90]
Multidrug resistance protein 1	37**-F**SMFR**Y**SN**W**-45	Yes	No	[42]
Neurofibromin	1606-**F**Y**Y**VARR**F**-1613	No	−	[91]
	1658-**F**LSKW**F**VV**F**-1666	No	−	
	1678-**Y**I**Y**NCNS**W**-1685	No	−	
	2102-**Y**L**F**HVVF**F**-2109	No	−	
nNOS	584-**F**SACP**F**SG**W**-592	Yes	No	[43]
iNOS	364-**F**PGCP**F**NG**W**-372	No	−	[92]
eNOS	348-**F**PAAP**F**SG**W**-356	Yes	No	[93]
PDGFR-α	879-**W**S**Y**GILL**W**-886	No	−	[94]
PDGFR-β	887-**W**S**F**GILL**W**-894	No	−	[94]
PDK1	141-**F**FVKL**Y**FT**F**-149	Yes	No	[37]
	299-**Y**D**F**PEKF**F**-306	Yes	No	
PP-1A	144-**Y**NIKL**W**KT**F**-152	No	−	[95]
PP2A-β	143-**W**K**Y**FTDL**F**-150	No	−	[95]
Protein kinase Cα	522-**W**A**Y**GVLL**Y**-529	No	−	[96]
	656-**F**S**Y**VNPQ**F**-663	No	−	
Protein kinase Cγ	539-**W**S**F**GVLL**Y**-546	No	−	[97]
	673-**F**T**Y**VNPD**F**-680	No	−	
Protein kinase Cζ	428-**Y**G**F**SVDW**W**-435	No	−	[96]
Ptc	788-**Y**D**F**IAAQ**F**KY**F**-798	Yes	No	[98]
PTEN	271-**F**H**F**WVNT**F**-278	Yes	No	[39]
PTPN1	174-**F**HYTT**W**PD**F**-182	No	−	[99]
PTPN6	206-**F**V**Y**LRQP**Y**-213	No	−	[99]
PTPN11	420-**W**Q**Y**HFRT**W**-427	No	−	[99]
Recoverin	65-**Y**AQHY**F**RS**F**-73	No	−	[100]
Rho-associated protein kinase 1	135-**W**VVQL**F**CA**F**-143148-**Y**L**Y**MVME**Y**-155	No	−	[101]
Rho-related GTP binding protein RhoC	34-**Y**VPTV**F**EN**Y**-42	No	−	[102]
Sialidase-3	179-**Y**T**Y**YIPS**W**-186	Yes	No	[36]
SKR3	399-**W**A**F**GLVL**W**-406	Yes	No	[32]
Slo1	1130-**Y**NMLC**F**GI**Y**-1138	Yes	Yes (Sucrose gradient)	[30], [44]
Sodium/calcium exchanger 1	259-**Y**KYV**Y**KR**Y**-266	No	−	[103]
	654-**Y**L**F**GQPV**F**-661	No	−	
Sodium/potassium-transporting ATPase subunit alpha-1	92-**F**CRQL**F**GG**F**-100987-**W**W**F**CAFP**Y**-994	Yes	No	[104], [105]
Solute carrier family 22 member 11	158-**F**I**W**GLLS**Y**-165	No	−	[106]
Solute carrier family 22 member 8	216-**Y**C**Y**TFGQ**F**-223	No	−	[107]
Striatin	55-**F**LQHE**W**AR**F**-63	No	−	[108]
Striatin-4	71-**F**IQHE**W**AR**F**-79	No	−	[108]
Sulphonylurea receptor 2B	138-**F**L**Y**WVMA**F**-145	No	−	[109]
TLR4	1146-**F**Y**F**IQKY**F**-1153	No	−	[110]
TNF receptor associated factor 2	354-**F**I**W**KISD**F**-361	No	−	[111]
Transforming protein RhoA	34-**Y**VPTV**F**EN**Y**-42	No	−	[22]
TrpC1	781-**F**RTSK**Y**AM**F**-789	Yes	No	[84], [112]
Type-1 angiotensin II receptor	302-**Y**G**F**LGKK**F**KR**Y**-312	Yes	No	[61], [113]
VEGFR-2	1089-**W**S**F**GVKK**W**EI**F**-1099	No	−	[114]
VEGFR-3	1098-**W**S**F**GVLL**W**EI**F**-1108	No	−	[115]

Clearly, the existence of a putative CBM sequence in a protein which binds to caveolin offers no guarantee of its involvement in binding. Nevertheless, as previously discussed, there are several examples where mutagenesis of the putative CBM leads to altered behaviour, although these cases are in the minority (Table 1). Unfortunately, it is not common practice to verify the folded state of the mutant protein. We consider below (see later) whether mutation of aromatic residues in putative CBM sequences may affect function through destabilisation of the protein fold, rather than the binding role often inferred.

**Table 2:** List of Cav-1-interacting molecules reported as containing CBM-like motifs.

Caveolin associated molecule	CBM location	References
Androgen Receptor	739-**Y**S**W**MGLMV**F**ANG**W**RS**F**-754	[116]
ATP-binding cassette sub-family G member 2	571-**F**SIPR**Y**G**F**-578	[117]
Beta-adrenergic receptor kinase 1	63-LGYLL**F**RD**F**-71	[76]
Calcium release-activated calcium channel protein 1	52-**Y**PD**W**IGQS**Y**-60	[118]
Desmoglein-2	776-**F**TDKAAS**Y**-783	[119]
ESR1	52-**Y**N**Y**PEGAA**Y**-60	[120]
	89-**F**GSNGLGG**F**-97	
Furin	742-**F**S**F**RGVKV**Y**-750759-**Y**KGLPPEA**W**-767	[121]
Gap junction alpha-1 protein	25-**W**LSVLFI**F**-32	[122]
Haem oxygenase-1	227-**F**LLNIQL**F**-234	[40]
Inositol 1,4,5-trisphosphate receptor type 3	1413-**Y**VN**F**VNHC**Y**-1421	[84]
Insulin-like growth factor-binding protein 5	240-IC**W**CVDK**Y**-247	[123]
LMW-PTP	78-ITKED**F**AT**F**-86	[23]
MAL-like protein	79-**F**G**F**YKR**F**-85	[89]
PPAR-gamma	360-**F**GD**F**MEPK**F**E**F**370	[124], [125]
Prostacyclin synthase	99-**Y**AI**F**LMER**IF**-108171-**F**LLRAG**Y**LTL**Y**-181	[126]
Protein kinase Cα	399-**F**LTQLHSC**F**-407	[127]
PTPRF	541-IM**Y**ELVY**W**-548	[99]
	1355-**F**T**W**ENSNL-1362	
Slo1	602-**Y**TE**Y**LSSA**F**-610	[30]
Solute carrier family 22 member 8	246-**F**FV**F**FLSS**W**-255	[107]
TGF-beta receptor type-1	424-**Y**QLPY**Y**DLV-433	[128]
	388-INMKH**F**ES**F**-396	
	393-**F**ES**F**KRADI**Y**-402	
TNF-receptor superfamily member 6	53-HHDGQ**F**CHH-62	[129]
Voltage-dependent anion-selective channel protein 1	62-**Y**R**W**TEYGL-69	[130]

### CBM Sequences are Abundant in the Human Proteome

The more specific a motif, the less frequently it will arise by chance during evolution. Conversely, very simple motifs will arise frequently by chance so that discovering that one is commonly found among a group of functionally related proteins – the caveolin interactome, for example – becomes less significant in itself. We therefore searched the human proteome for the CBM motifs. Strikingly, this analysis shows that ∼30% of all proteins contain at least one instance of a **ф**X**ф**XXXX**ф** or **ф**XXXX**ф**XX**ф** sequence. This number increases to 69% by allowing substitution of either I or L at one of the aromatic positions. It is highly likely that the majority of these proteins have no interaction with caveolin and that many proteins possess putative CBMs by chance. Consequently, identification of a CBM within a protein may not be strong evidence to suggest a direct interaction with caveolin.

### Aromatic-containing Motifs are not Significantly Enriched in the Caveolin Interactome

The high frequency of the CBM motifs in the human proteome does not, of course, mean that they may not serve in some proteins for interaction with caveolin. If that were the case, a statistically higher occurrence of the CBM motifs in the caveolin interactome, compared to proteins in general, would be expected. We therefore used the web-based short linear motif (SLiM) discovery service, SLiMFinder, to search for any over-represented motifs (CBM-like or novel) among the Cav-1 interactome. The complete Cav-1 interactome used in this study can be found as supporting data (Table S1). SLiMFinder is a probabilistic web server program for identification of SLiMs in proteins with a common attribute (such as a common interaction partner) and for estimating the probability of returned motifs arising by chance [25], [45]. Caveolin 1 was chosen for this analysis since, compared to the other two isoforms, it has the most abundant interaction data. The available interactome data for Cav-2 and Cav-3 was considered too small to derive statistically meaningful information and was therefore not included in this study. The sequences of 135 proteins with multiple experimentally-demonstrated interactions with Cav-1 were collected by surveying databases such as IntAct v.3.1, BioGrid^3.1^, and APID-beta and from the literature. The SLiMFinder web-server was run on this dataset, altering search parameters in order to ensure that motifs matching the original CBM definitions would be returned if statistically significantly enriched. SLiMFinder returned just one SLiM ([ST].[LV]$; where $ represents the C-terminus) below the default significance threshold of 0.05 [45]. This was present in only 11 proteins and is an already known motif (LIG_PDZ_Class_1 in the ELM database [46]) specifying interaction with PDZ domains. Even restricting the dataset to 64 proteins identified in the literature to contain a CBM, failed to return any motifs resembling the CBMs. Furthermore, CBM-like or aromatic-rich motifs were not returned for either data set even at higher, non-significant e-values (up to a threshold cut-off of 0.99).

As SLiMs tend to occur in disordered regions of proteins [47], the SLiMFinder webserver, by default, masks out regions predicted to be ordered by IUPred [48] which thus excludes them from further analysis and improves performance. Consequently, CBMs which occur in domains with predicted higher order (e.g. the tyrosine kinase domain of insulin receptor [49] and catalytic domain of protein kinase A [14]) are likely removed from the motif discovery process. To see if their inclusion affected motif discovery, disorder masking was deactivated and a SLiMFinder run was repeated for the datasets. However, CBM-like motifs were once again absent from the list of statistically significant and insignificant motifs. This suggests that the aromatic-rich CBMs are not statistically over-represented in proteins known to interact with Cav-1.

### CBMs Identified in the Literature Lack the Characteristics of SLiMs

Most SLiMs share a set of characteristics including a tendency to be located in surface accessible intrinsically disordered regions, a high degree of conservation relative to the local background sequence, and a tendency to contain residues with greater likelihood to undergo order-disorder transitions [45], [47]. It is therefore possible to computationally predict regions where motifs are likely to occur from a protein's primary sequence. We therefore applied SLiMPred, a recent *de novo* web-based programme designed to predict SLiMs from both ordered and disordered protein sequences independently of experimentally defined homologues and interactors [27], to see if putative CBMs coincide with regions predicted to have these SLiM-like characteristics. The analysis was limited to include only proteins with experimental evidence to suggest that the CBM is involved in binding to caveolin. The SLiMPred algorithm bases its predictions on annotated instances from the Eukaryotic Linear Motif database, as well as structural, biophysical, and biochemical features derived from the protein's primary sequence, and assigns each residue of a protein with a probability value between 0 and 1, with residues scoring closer to 1 most likely belonging to a SLiM. A threshold for residues to be considered a SLiM residue was set at 0.1, at which there exists a balance between a reasonable true-, and a low false-positive rate (44 and 22% respectively [27]). Values for CBM aromatic residues are listed in Table 3. Even with such a low cut off point, only ∼36% of CBM aromatic residues were predicted to be part of a motif and thus able to facilitate protein-protein interactions (Table 3). In only four of 28 CBM cases were all three aromatic residues so predicted, while in 13 cases none of the three aromatic residues gave a positive prediction. Furthermore, of the CBM aromatic residues which were scored highly by SLiMPred, 68% coincide with known functional motifs unrelated to caveolin binding. For example, SLiMPred matched Y786 and F789 of the short transient receptor potential channel 1 (TrpC1) CBM to the previously discovered TRG_ENDOCYTIC_2 motif, which is a tyrosine-based sorting signal responsible for interaction with the mu-subunit of the AP (adaptor protein) complex. It is not however known whether this is a functional motif for TrpC1. SLiMPred scores for the entire stretch of CBM residues, including non-defined and non-functional positions, are available as supplementary information (Table S2). Overall, these tests indicate that most published examples of CBMs in proteins binding caveolin lack the characteristics of known functional SLiMs.

**Table 3:** Probability of a CBM aromatic residue belonging to a SLiM.

Caveolin associated molecule	SLiMPred score			
ABPP	Y757 0.07	**Y762 0.25**	**F764 0.29**	−
Beta-adrenergic receptor kinase 1	W576 0.00	**F581 0.125**	F584 0.00	−
Btk	W581 0.00	F583 0.05	W588 0.00	−
D(1A) Dopamine receptor	**F313 0.13**	**W318 0.13**	**W321 0.11**	−
EGFR	W898 0.00	Y900 0.00	W905 0.00	−
Ephrin type-B receptor 1	W808 0.00	Y810 0.00	W815 0.00	−
Gi2 subunit-α	F190 0.00	F192 0.00	F197 0.00	−
Glucagon-like peptide 1 receptor	Y250 0.09	Y252 0.09	**F257 0.10**	−
Insulin receptor	W1220 0.00	F1222 0.00	W1227 0.00	−
Integrin-linked protein kinase	W376 0.00	F378 0.00	W383 0.06	−
Metabolic glutamate receptor 1	**F609 0.46**	F614 0.05	**Y617 0.12**	−
	F781 0.08	**Y786 0.15**	**Y789 0.11**	−
Multidrug resistance protein 1	**F37 0.43**	**Y42 0.33**	**W45 0.17**	−
nNOS	F584 0.00	F589 0.00	W592 0.00	−
eNOS	F348 0.00	F353 0.00	W356 0.00	−
PDK1	F141 0.00	Y146 0.00	F149 0.00	−
	Y299 0.08	F301 0.09	F306 0.01	−
Ptc	Y788 0.00	**F790 0.46**	**F795 0.56**	**F798 0.51**
PTEN	F271 0.00	F273 0.00	F278 0.00	−
Sialidase-3	Y179 0.00	Y181 0.00	W186 0.00	−
SKR3	W399 0.00	F401 0.00	W406 0.04	−
Slo1	Y1130 0.05	**F1135 0.18**	Y1138 0.02	−
Sodium/potassium-transportingATPase subunit alpha-1	**F92 0.10** **W987 0.39**	F97 0.05**F989 0.55**	F100 0.08**Y994 0.40**	−
Striatin	**F55 0.42**	**W60 0.15**	**F63 0.25**	−
TLR4	F741 0.00	**W746 0.18**	F749 0.09	−
TrpC1	**F781 0.12**	**Y786 0.53**	**F789 0.33**	−
Type-1 angiotensin II receptor	**Y302 0.11**	F304 0.00	F309 0.05	**Y312 0.12**

SLiMPred webserver was run on proteins with experimental evidence suggesting the CBM facilitates binding to Cav-1. Predicted SLiM residues (SLiMPred score>0.1) are in bold. SLiMPred scores for non-functional CBM residues are available as supporting data (Table S2).

### CBM Aromatic Residues are Mostly Unavailable for Caveolin Interaction

**Figure 1:**
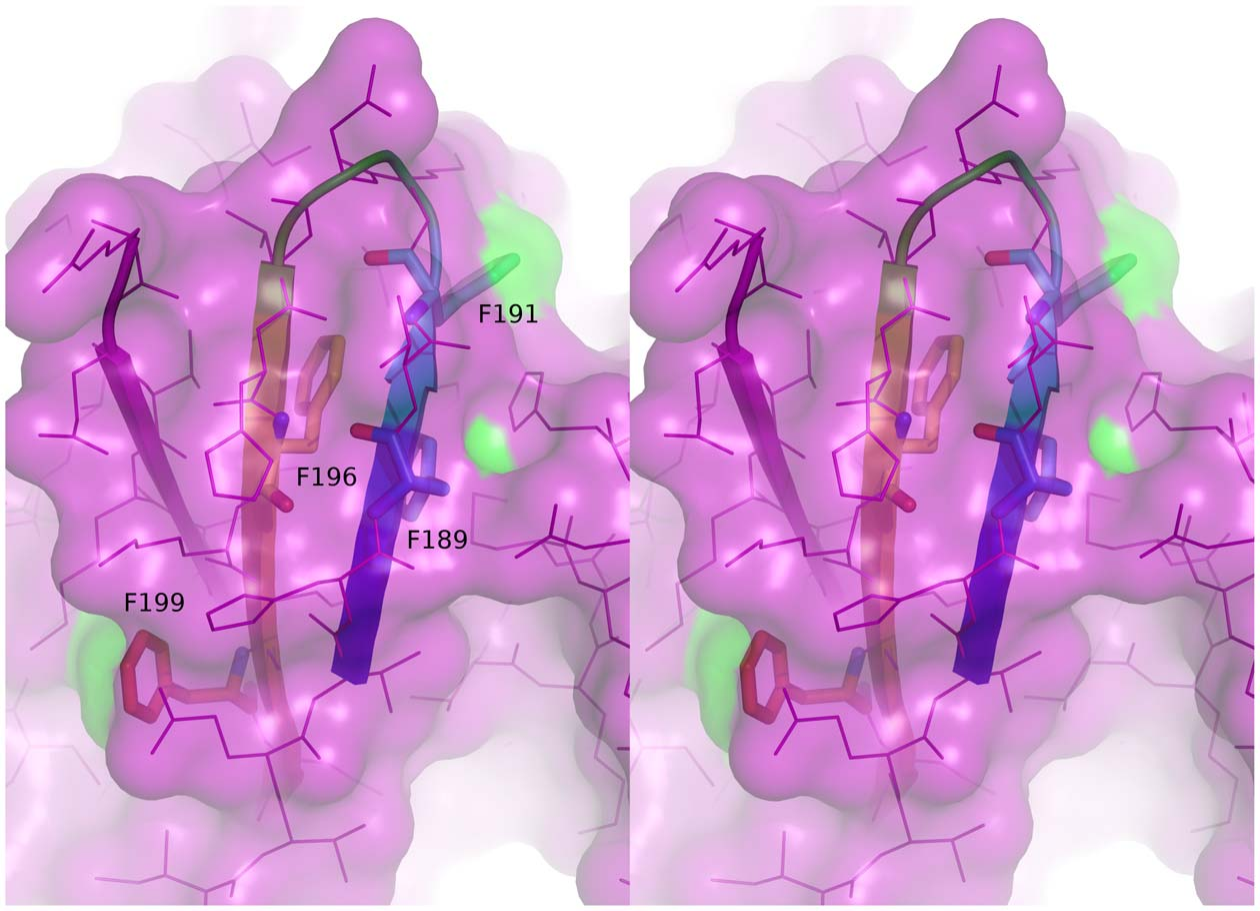
Cross-eyed stereo view of the context of the CBM of Couet *et al* [**6**] seen in the rat Gi1α protein (PDB code 1CIP; [**51**]). The β-hairpin structure of the motif is shown as a cartoon, coloured from blue to red, and the aromatic residues drawn as sticks (Phe189 is blue, Phe191 is cyan, Phe196 is yellow and Phe199 is red). The third strand of the three-stranded sheet to which the motif belongs is also shown in pink. The remainder of the protein is shown as lines and surface, the latter coloured green where contributed by side chains of the aromatic residues.

The aromatic residues of the defined CBMs are largely hydrophobic, especially Phe, and so are most commonly found buried in the structural core of proteins. Surface exposure of such residues to allow interaction with other molecules is known, as in carbohydrate-binding proteins for example [50], but is uncommon. For the CBM sequence, and specifically the aromatic residues, to function *in situ* within the Gi2α protein for binding caveolin (as first described by Couet *et al.* [6]), it and they must be accessible for interaction. The nearest relatives of the Gi2α protein with known structures are rat and human Gi1α sequences, which are sequence-identical in the vicinity of the CBM. Figure 1 shows the position of the CBM in the highest resolution structure of a native Gi1α sequence (rat Gi1α PDB code 1CIP; [51]). The motif adopts a β-hairpin structure, extensively hydrogen bonded to a third strand. Of the four aromatic positions, only the second and fourth are significantly solvent-exposed, and their positions on opposite sides of the hairpin ensure that simultaneous interaction of both with caveolin is unlikely (Figure 1). Clearly, in the conformation captured by crystallography, two of the four aromatic residues are unavailable for inter-molecular interaction.

Although substantial conformational changes in the region are rendered unlikely by the embedding of the β-hairpin structure in a three strand β-sheet, we sought evidence that such a transformation is possible in two ways: by assessing conformational variability among other structures and by conformationally simulating the main modes of dynamics using an elastic network model [52]. Figure S1 shows a comparison of all available rat and human Gi1α structures in the CBM region, showing that the position of the aromatic residues is essentially the same in each. Figure S2 shows the same region in a broader selection of G proteins in which at least three of the four aromatic positions are present. Again, the β-hairpin and three-stranded sheet are structurally conserved and where aromatic residues are found at positions corresponding to those in Gi1α they are similarly generally buried. Finally, we predicted the major conformational modes of Gi1α using the AD-ENM server. None of the largest 10 predicted motions impacts significantly on the CBM and the β-hairpin. For illustration, the motion leading to the largest structural variation in the motif region (eigenvector 8) is shown in Figure S3 where its maximum and minimum projections are superimposed on the crystal structure. Once again the hydrogen-bonding between the β-hairpin and third strand is stable ensuring that all aromatic residues maintain similar, largely buried conformations. Side chains are not treated by the AD-ENM analysis. These considerations lead us to conclude that it is difficult to imagine interaction of CSD with the CBM in Gi2α, as visualised crystallographically, involving more than one or two of its aromatic residues. Furthermore, there is no apparent support for the idea that the region is particularly conformationally flexible and thus capable of adopting radically different structures in which multiple aromatics would be suitably exposed and arrayed for interaction with the CSD. Moreover, the crystal structures of other known caveolin binding proteins with proposed functional CBMs (EGFR, insulin receptor, integrin-linked protein kinase, PDK1, PTEN and Slo1) also suggest that CBM residues are largely buried (Figure S4).

To see how general an issue accessibility could be for the CBM hypothesis, we measured solvent accessibility of aromatic residues in CBMs of Cav-1 interacting proteins *in situ*. Experimental structures of the proteins were preferentially used for this analysis. For proteins where structures were unavailable, homology models were used if template availability allowed. The relative solvent accessible areas (RSAs) of the CBM aromatic residues were calculated as previously described ([53]; see Methods for details) and are listed in Table 4. It is worth noting that these values will in some cases be overestimates of solvent accessibility since some experimental structures will be of isolated domains, not complete proteins, and some homology models may also be incomplete. For instances where no structure was available, SABLE [54] was used to estimate the RSA (Table 5). The resulting data (Tables 4 and 5) strongly indicate that the majority of CBM aromatic residues are buried (RSA<20% [53]) within the protein, and are thus unavailable for interaction directly with caveolin or with a third protein mediating an indirect interaction with caveolin. Notably, for the data set including experimental and model structures, only three out of 57 CBMs were predicted to contain three solvent-exposed aromatic residues, those of insulin-like growth factor-binding protein 3 (IBP-3), Kv1.3 and Kv1.5 (Table 4). Conversely, there are 25 CBM examples where all three aromatic residues are classified as buried. Table 4 also shows the secondary structure at each of the aromatic positions within the putative CBMs. It is notable that the secondary structure context varies widely, contrary to what would be expected if each of these sequences bound to caveolin in a similar manner.

**Table 4:** Relative exposed surface area (RSA) of CBM aromatics.

Caveolin associated molecule	Residue and RSA (%)				PDB code
ABPP	**Y757 17.4 (E)**	**Y762 12.6 (H)**	F764 20.0 (T)	−	3DXC
Aquaporin 1	W210 49.8 (G)	**F212 3.3 (H)**	F217 31.4 (H)	−	1H6I
Beta-adrenergic receptor kinase 1	W576 29.4 (-)	**F581 0.0 (E)**	**F584 0.0 (E)**	−	3CIK
Btk	**W581 0.0 (H)**	**F583 0.5 (H)**	**W588 7.1 (H)**	−	3GEN
Cytosolic phospholipase A2	**F683 4.3 (-)**	**Y685 0.0 (-)**	**F690 2.9 (H)**	−	1CJY
D(1A) Dopamine receptor	F313 31.0 (T)	W318 22.0 (H)	**W321 3.1 (G)**	−	1OZ5
EGFR	**W898 0.4 (H)**	**Y900 0.9 (H)**	**W905 6.3 (H)**	−	2J6M
Ephrin type-B receptor 1*	**W808 0.8 (H)**	**Y810 0.0 (H)**	**W815 1.6 (H)**	−	2SJS (T)
Fatty acid synthase*	**Y1506 7.4 (E)**	W1511 34.9 (E)	F1514 22.9 (−)	−	3HHD (T)
Fibroblast growth factor receptor 1	**W684 0.0 (H)**	**F686 0.0 (H)**	**W691 5.1 (H)**	**F694 3.3 (T)**	3GQL
Gi2 subunit-α*	**F190 2.9 (E)**	**F192 17.0 (E)**	**F197 1.0 (E)**	**F200 8.6 (E)**	1AGR (T)
Inositol 1,4,5-triphosphate receptor type 1*	**W218 0.4 (E)**	**F223 6.2 (E)**	W226 24.7 (H)	−	1XZZ (T)
Inositol 1,4,5-trisphosphate receptor type 3*	**W219 0.0 (E)**	**F224 0.1 (E)**	F227 20.5 (H)	−−	3T8S (T)
Insulin receptor	**W1220 1.2 (H)**	**F1222 0.0 (H)**	**W1227 6.3 (H)**	−	3BU3
Integrin-linked protein kinase	**W376 2.4 (H)**	**F378 0.0 (H)**	**W383 6.7 (H)**	−	3REP
IBP-3*	F261 60.0 (−)	W263 23.9 (−)	Y268 77.4 (T)	−	IH59 (T)
Kv1.3*	F216 28.1 (−)	W221 73.3 (T)	F224 71.4 (H)	−	3LUT (T)
Kv1.5*	F232 29.5 (−)	W237 72.5 (T)	F240 70.5 (H)	−	3LUT (T)
Leukemia inhibitory factor receptor	F323 23.8 (E)	**F328 7.6 (E)**	Y331 39.1 (−)	−	3E0G
Neurofibromin	**F1606 0.0 (E)**	**Y1608 11.3 (E)**	**F1613 0.0 (**−**)**	−	2EZX
	**F1658 6.7 (H)**	F1663 38.1 (T)	**F1666 12.9 (S)**	−	2EZX
	**Y1678 4.3 (E)**	**Y1680 0.0 (E)**	W1685 27.5 (H)	−	2EZX
nNOS*	**F584 0.0 (E)**	F589 24.0 (E)	W592 27.8 (−)	−	1VAG (T)
iNOS	**F364 0.0 (E)**	F369 22.0 (E)	W372 26.3 (−)	−	1NSI
eNOS	**F348 0.0 (E)**	F353 22.0 (E)	W356 29.0 (−)	−	1M9M
PDGFR-α	**W879 0.4 (H)**	**Y881 0.4 (H)**	**W886 10.2 (H)**	−	1GQ5
PDGFR-β	**W887 0.4 (H)**	**F889 1.4 (H)**	**W894 11.0 (H)**	−	1LWP
PDK1	**F141 1.9 (T)**	Y146 48.7 (E)	**F149 3.8 (E)**	−	1UU3
	**Y299 14.3 (**−**)**	**F301 11.4 (**−**)**	F306 21.9 (−)	−	1UU3
PP-1A	**Y144 14.8 (S)**	**W149 11.0 (H)**	**F152 0.0 (H)**	−	3E7A
PP2A-β*	**W143 8.2 (H)**	**Y145 13.9 (H)**	**F150 1.0 (H)**	−	2IE4 (T)
Protein kinase Cα	**W522 0.0 (H)**	**Y524 0.4 (H)**	**Y529 6.1 (H)**	−	3IW4
	**F656 0.0 (**−**)**	Y658 24.3 (E)	**F663 1.9 (**−**)**	−	3IW4
Protein kinase Cγ*	**W539 0.0 (H)**	**F541 0.5 (H)**	**Y546 5.2 (H)**	−	3PFQ (T)
	**F673 0.5 (**−**)**	Y675 35.2 (E)	F680 20.5 (−)	−	3PFQ (T)
Protein kinase Cζ*	**Y428 1.7 (B)**	**F430 18.6 (T)**	**W435 0.0 (H)**	−	3A8X (T)
PTEN	**F271 1.0 (E)**	**F273 1.4 (E)**	**F278 17.1 (G)**	−	1D5R
PTPN1	**F174 1.4 (E)**	**W179 1.2 (E)**	F182 58.6 (T)	−	1AAX
PTPN6	F206 46.2 (E)	Y208 39.6 (−)	**Y213 9.6 (**−**)**	−	2B3O
PTPN11	**W420 16.1 (E)**	**Y422 0.4 (E)**	**W427 7.8 (**−**)**	−	2SHP
Recoverin	**Y65 0.0 (H)**	**F70 0.0 (H)**	**F73 15.2 (H)**	−	2D8N
Rho-associated protein kinase 1	**W135 9.8 (T)**	F140 25.2 (E)	**F143 1.4 (E)**	−	2ESM
	**Y148 7.8 (E)**	**Y150 5.2 (E)**	**Y155 11.3 (**−**)**		
Rho-related GTP binding protein RhoC	Y34 60.4 (−)	**F39 3.8 (E)**	Y42 23.5 (E)	−	1Z2C
Sialidase-3*	**Y179 9.6 (E)**	Y181 22.6 (E)	W186 72.2 (T)	−	2F25 (T)
SKR3	**W399 0.8 (H)**	**F401 1.9 (H)**	**W406 3.9 (H)**	−	3MY0
Slo1	Y1130 49.0 (S)	**F1135 4.8 (E)**	**Y1138 3.0 (E)**	−	3MT5
Sodium/calcium exchanger 1*	Y654 50.4 (H)	F656 24.8 (H)	**F661 17.6 (H)**	−	2FWU (T)
Sodium/potassium-transporting ATPase subunit alpha-1*	F92 34.8 (T)**W987 9.0 (T)**	F97 59.5 (T)F989 47.6 (T)	**F100 4.3 (T)**Y994 25.7 (H)	−−	3B8E (T)
Sulphonylurea receptor 2B *	**F1146 6.2 (H)**	**F1148 2.8 (**−**)**	**F1153 4.9 (**−**)**	−	2CBZ (T)
TNF receptor associated factor 2	**F354 1.9 (E)**	**W356 9.4 (E)**	**F361 3.8 (H)**	−	1CA9
TLR4*	**F741 0.5 (H)**	**W746 7.8 (S)**	**F749 10.0 (H)**	−	3J0A (T)
Transforming protein RhoA	Y34 24.3 (−)	**F39 16.2 (E)**	Y42 27.0 (E)	−	3MSX
Type-1 angiotensin II receptor	**Y302 14.3 (H)**	F304 68.1 (S)	**F309 2.6 (H)**	**Y312 15.7 (H)**	1ZV0
VEGFR-2	**W1089 0.4 (H)**	**F1091 0.0 (H)**	**W1096 6.0 (H)**	−	3EWH
VEGFR-3*	**W1098 0.0 (H)**	**F1100 0.0 (H)**	**W1105 5.5 (H)**	**F1108 0.0 (T)**	2VHE (T)
					

**Table 5:** SABLE estimates of relative exposed surface area (RSA) of CBM aromatics.

Caveolin associated molecule	Residue and RSA (%)			
Adenosine receptor A1	**Y288 0**−**9**	**F290 0–9**	**F296 0–9**	−
cGMP-inhibited 3',5'-cyclic phosphodiesterase B	F47 20–29**W330 10–19**	**F49 10–19** **W332 10–19**	F54 20–29W337 20–29	−−
Sulphonylurea receptor 2B	**F138 0–9**	**Y140 0–9**	**F145 0–9**	−
Glucagon-like peptide 1 receptor	**Y250 0–9**	**Y252 0–9**	F257 20–29	−
Inositol 1,4,5-triphosphate receptor type 1	**Y2461 0–9**	**F2463 0–9**	**Y2468 0–9**	−
Interleukin-6 receptor subunit beta	F606 30–39	**F608 10–19**	F613 40–49	−
MAL-like protein	**F23 10–19**	**F28 0–9**	**F31 0–9**	−
	**Y143 0–9**	**F148 0–9**	**Y151 0–9**	−
Metabolic glutamate receptor 1	**F609 0–9**	**F614 0–9**	**Y617 10–19**	−
	**F781 10–19**	**Y786 10–19**	**Y789 0–9**	−
Metalloreductase STEAP4	**Y192 0–9**	**F197 10–19**	**W200 10–19**	−
	**Y271 10–19**	**F276 10–19**	**F279 0–9**	−
Multidrug resistance protein 1	**F37 10–19**	**Y42 0–9**	**W45 20–29**	−
Neurofibromin	Y2102 20–29	**F2104 0–9**	**F2109 0–9**	−
Ptc	**Y788 10–19**	**F790 0–9**	F795 30–39	**F798 10–19**
Sodium/calcium exchanger 1	Y259 20–29	Y261 20–29	Y266 20–29	−
Solute carrier family 22 member 11	**F158 0–9**	**W160 0–9**	**Y165 10–19**	−
Solute carrier family 22 member 8	**Y216 0–9**	**Y218 0–9**	**F223 0–9**	−
Striatin	**F55 0–9**	**W60 10–19**	**F63 10–19**	−
Striatin-4	**F71 0–9**	**W76 10–19**	F79 20–29	−
TrpC1	F781 40–49	**Y786 10–19**	F789 20–29	−

Predictions are given in ranges spanning 10%. Buried residues (RSA<20%) are in bold font.

The burial of CBM aromatic positions, rendering them unavailable for interaction, apparently conflicts with the findings of the numerous authors discussed earlier who demonstrate that CBM mutation severely disrupts protein interactions with caveolin. However, in these examples, data are very rarely presented to demonstrate that the protein folding is unaffected by the mutation. This offers an alternative explanation for situations in which aromatic residues are buried and unavailable for interaction yet their mutation affects interaction with caveolin: the aromatic residues are critical for protein stability [53] and their mutation leads to destabilisation of the protein fold and knock-on effects on the caveolin interface. We used PoPMuSiC, which accurately predicts values of ΔΔG free energy stability change resulting from point mutations [55], [56], to anticipate the potentially deleterious effects of CBM aromatic substitution with alanine, the most common mutation experimentally chosen. In nearly all cases, mutation of a buried CBM aromatic had a predicted significant destabilizing effect on the protein (>2.0 kcal/mol; Table 6 [57]). Considering that the majority of CBM aromatics are buried, it is likely that experimental mutation of these residues would impair protein stability, which may explain observed abrogation of caveolin interaction in some or even most cases. Indeed, most CBMs are highly conserved in sequence (Table S3) consistent with the idea that their aromatic residues are important determinants of protein structure.

**Table 6:** Predicted change in folding free energy (ΔΔG) resulting from alanine mutation.

Caveolin associated molecule	Residue and ΔΔG (kcal/mol)				PDB code
ABPP	**Y757 2.49**	**Y762 2.28**	**F764 2.70**	−	3DXC
Aquaporin 1	W210 0.84	**F212 2.70**	F217 1.43	−	1H6I
Beta-adrenergic receptor kinase 1	**W576 2.40**	**F581 3.60**	**F584 3.89**	−	3CIK
Btk	**W581 3.72**	**F583 2.78**	**W588 2.90**	−	3GEN
Cytosolic phospholipase A2	**F683 3.30**	**Y685 3.41**	**F690 3.08**	−	1CJY
D(1A) Dopamine receptor	F313 1.59	**W318 2.46**	**W321 3.33**	−	1OZ5
EGFR	**W898 3.87**	**Y900 3.01**	**W905 2.86**	−	2J6M
Ephrin type-B receptor 1*	**W808 3.89**	**Y810 3.00**	**W815 3.45**	−	2SJS (T)
Fatty acid synthase*	**Y1506 3.10**	W1511 1.79	**F1514 2.17**	−	3HHD (T)
Fibroblast growth factor receptor 1	**W684 3.77**	**F686 2.57**	**W691 2.94**	**F694 2.80**	3GQL
Gi2 subunit-α*	**F190 3.18**	**F192 3.43**	**F197 4.02**	**F200 2.92**	1AGR (T)
Inositol 1,4,5-triphosphate receptor type 1*	**W218 3.78**	**F223 3.34**	**W226 2.48**	−	1XZZ (T)
Inositol 1,4,5-trisphosphate receptor type 3*	**W219 3.71**	**F224 3.08**	**F227 2.45**	−	3T8S (T)
Insulin receptor	**W1220 3.97**	**F1222 3.08**	**W1227 2.90**	−	3BU3
Integrin-linked protein kinase	**W376 3.15**	**F378 2.54**	**W383 2.99**	−	3REP
IBP-3*	F261 0.65	**W263 2.21**	Y268 0.17	−	IH59 (T)
Kv1.3*	F216 1.84	W221 −0.03	F224 0.36	−	3LUT (T)
Kv1.5*	F232 1.75	W237 0.23	F240 0.23	−	3LUT (T)
Leukemia inhibitory factor receptor	**F323 2.07**	**F328 2.71**	Y331 1.61	−	3E0G
Neurofibromin	**F1606 3.29**	**Y1608 2.85**	**F1613 3.49**	−	2EZX
	**F1658 2.56**	F1663 1.59	**F1666 2.78**	−	2EZX
	**Y1678 2.94**	**Y1680 4.08**	W1685 1.96	−	2EZX
nNOS*	**F584 3.79**	**F589 3.28**	**W592 2.81**	−	1VAG (T)
iNOS	**F364 3.64**	**F369 2.67**	**W372 2.98**	−	1NSI
eNOS	**F348 3.31**	**F353 3.63**	**W356 2.72**	−	1M9M
PDGFR-α	**W879 3.44**	**Y881 3.01**	**W886 2.97**	−	1GQ5
PDGFR-β	**W887 3.76**	**F889 2.87**	**W894 2.84**	−	1LWP
PDK1	**F141 3.44**	Y146 0.68	**F149 3.13**	−	1UU3
	**Y299 3.29**	**F301 3.06**	F306 1.91	−	1UU3
PP-1A	**Y144 2.79**	**W149 3.25**	**F152 3.03**	−	3E7A
PP2A-β*	**W143 2.53**	**Y145 2.49**	**F150 2.63**	−	2IE4 (T)
Protein kinase Cα	**W522 4.10**	**Y524 3.43**	**Y529 2.48**	−	3IW4
	**F656 3.04**	Y658 1.86	**F663 2.88**	−	3IW4
Protein kinase Cγ*	**W539 3.78**	**F541 3.18**	**Y546 2.44**	−	3PFQ (T)
	**F673 3.10**	Y675 1.68	**F680 2.28**	−	3PFQ (T)
Protein kinase Cζ*	**Y428 3.39**	**F430 2.33**	**W435 4.18**	−	3A8X (T)
PTEN	**F271 4.17**	**F273 3.38**	F278 1.97	−	1D5R
PTPN1	**F174 3.94**	**W179 3.95**	F182 0.85	−	1AAX
PTPN6	F206 1.11	Y208 1.45	**Y213 3.23**	−	2B3O
PTPN11	**W420 3.25**	**Y422 3.32**	**W427 3.49**	−	2SHP
Recoverin	**Y65 3.08**	**F70 3.15**	**F73 2.35**	−	2D8N
Rho-associated protein kinase 1	**W135 3.54**	**F140 2.03**	**F143 3.49**	−	2ESM
	**Y148 3.53**	**Y150 3.42**	**Y155 2.84**	−	
Rho-related GTP binding protein RhoC	Y34 0.51	**F39 3.48**	**Y42 2.45**	−	1Z2C
Sialidase-3*	**Y179 3.43**	**Y181 2.18**	W186 0.18	−	2F25 (T)
SKR3	**W399 3.75**	**F401 3.15**	**W406 3.05**	−	3MY0
Slo1	Y1130 0.69	**F1135 2.99**	**Y1138 3.56**	−	3MT5
Sodium/calcium exchanger 1*	Y654 0.79	F656 1.26	F661 1.87	−	2FWU (T)
Sodium/potassium-transporting ATPase subunit alpha-1*	F92 1.57**W987 3.44**	F97 0.75F989 1.32	**F100 2.59**Y994 1.82	−−	3B8E (T)
Sulphonylurea receptor 2B *	**F1146 2.21**	**F1148 3.17**	**F1153 4.60**	−	2CBZ (T)
TLR4*	**F741 3.81**	**W746 3.36**	**F749 2.74**	−	3J0A (T)
TNF receptor associated factor 2	**F354 3.44**	**W356 3.58**	**F361 2.84**	−	1CA9
Transforming protein RhoA	**Y34 2.30**	**F39 2.59**	**Y42 2.21**	−	3MSX
Type-1 angiotensin II receptor	**Y302 2.56**	Y304 −0.10	**F309 2.50**	**Y312 3.18**	1ZV0
VEGFR-2	**W1089 3.86**	**F1091 2.63**	**W1096 3.14**	−	3EWH
VEGFR-3*	**W1098 4.22**	**F1100 2.97**	**W1105 3.04**	**F1108 3.43**	2VHE (T)

Predictions calculated using PoPMuSiC. ΔΔG values for proteins marked with * were determined from homology models (SWISS MODEL repository) and the PDB code given is that of the template (T) used for the model. Mutations predicted as significantly destabilising (ΔΔG>2.00 kcal/mol) in bold font.

Some experimental data support this idea. For example, mutations of insulin receptor CBM aromatics result in poorly expressed mature constructs at the cell surface, impaired autophosphorylation, and accelerated degradation of the proreceptor [41], [58]–[60] which is consistent with the notion of buried aromatics being important structural factors. F313A and W318A mutation of the putative D(1A) dopamine receptor CBM resulted in a protein with similar pharmacological properties and surface expression as the wild-type receptor, but which had lost its ability to bind to Cav-1 [33]. Whereas these two amino acids are relatively exposed (RSAs of 31 and 22% respectively; Table 4) and may contribute to a real binding site for caveolin, the final aromatic position of this CBM, W321, is deeply buried (RSA = 3%) and its mutation to alanine is consequently predicted to have the strongest destabilizing effect of the three aromatics (3.33 kcal/mol). Accordingly, Kong *et al.* [33] reported that the W321A mutant exhibited strongly attenuated surface expression and pharmacological activity, indicative of protein misfolding. Thus, it is unlikely that all three of the CBM aromatics participate in the interaction with caveolin. Furthermore, mutation of nNOS F589 and W592 residues to Leu only partially abrogates interaction with Cav-1 [43]. Suggestively, such mutations are predicted to have a less severe destabilising effect (1.04 and 1.36 kcal/mol for F589L and F592L respectively) than mutation to alanine, which may explain the retained Cav-1 binding.

Although we assert that the general burial of putative CBMs in known and model structures argues against their having functionality, there is the possibility of CBM sequences exerting their function before the protein in which they are embedded achieves its final conformation. Thus, Wyse *et al*. [61] demonstrated that, despite not forming a complex with caveolin in the caveolae, expression of Cav-3 and an intact CBM of type 1 receptor for angiotensin II (AT1-R) are critical for the correct trafficking and localisation of the receptor to the cell surface, as AT1-R is found exclusively in the ER in caveolin-deficient cells and following mutation of each CBM aromatic. This was explained by Cav-3 binding to AT1-R during the initial stage of AT1-R maturation in the ER, and serving as a chaperone to shuttle the receptor to the plasma membrane. Although only one of the CBM aromatics is exposed in the mature receptor (F304; Table 4), the CBM as a whole may be in a more accessible conformation within the ER before the receptor reaches its final natively folded structure. Caveolin has also been identified as a transport chaperone for glycosylphosphatidylinositol-anchored proteins, which are only surface expressed in the presence of Cav-1 or Cav-3 [62]. Interestingly, the CBM is reminiscent of another possible motif recognised by the chaperone BiP, found in the endoplasmic reticulum. The BiP recognition motif is Hy(W/X)HyXHyXHyXHyX, where Hy is a large hydrophobic amino acid, most frequently Trp, Leu, or Phe, and X is any amino acid. The comparison was already made by Couet *et al.* [6] but they argued for a role as a 'membrane chaperone' whereas the data published since opens up the possibility of caveolin functioning in the ER en route to the plasma membrane. This potential chaperone aspect of caveolin function clearly merits further investigation.

## Discussion

Since the original definition of the CBM was proposed by Couet *et al*. [6] the notion of these aromatic-rich motifs has become firmly embedded in the literature. However, since these early experiments, greater structural information has become available for potential caveolin binding proteins. Taking advantage of this and recent advances in bioinformatics methodologies, we have critically evaluated the perceived role of the CBM as the dominant site for caveolin association. The web-based algorithms, SLiMFinder and SLiMPred, did not recognise the CBMs of caveolin binding proteins as functional motifs which could facilitate protein-protein interactions directly or indirectly with caveolin. Furthermore, a complete CBM is rarely expressed at the surface of a protein as the bulk of CBM aromatics are buried and as such would be an unsuitable interface for protein binding. The often demonstrated requirement for an unperturbed CBM for caveolin binding may instead relate to its function – and in particular the role of the aromatic residues – in determining the structure of caveolin target proteins.

As the aromatic residues of Gi2α protein derived peptides were not individually mutated in the original work of Couet *et al.* [6], there is little reason to suppose that a motif of this arrangement would invariably be required for caveolin binding. In this regard it is noteworthy that many authors have presented evidence to suggest that proteins lacking CBMs or with incomplete or CBM-like motifs interact with caveolin. It is interesting that the CSD and the predicted consensus for caveolin binding motifs are both aromatic-rich sequences. In the original experiments of Couet *et al*. [6], binding of the CSD with the aromatic-rich Gi2α protein derived peptides would likely have been due to π-stacking of aromatic amino acid side chains. Therefore, although the concept of a traditional CBM, where all three aromatic residues are a necessity for caveolin interaction, may not be physiologically relevant due to residue inaccessibility, these early experiments indicate that the CSD may have high propensity for hydrophobic and π-stacking interactions. For example, Yue & Mazzone [63] observed that human apoE is enriched in aromatic amino acids in a non-CBM configuration between residues 44 and 63, and demonstrated that a biotin-labelled peptide of 20 residues containing this region binds Cav-1 from adipocyte lysates. Furthermore, in a CSD-PKAcat structural model, the CSD is predicted to extend across PKAcat and make contacts with several surface-located hydrophobic and aromatic residues (P244, I245, Y248) in addition to hydrogen bonding interactions [64].

In summary, we argue that the notion of aromatic-containing CBMs has taken an unwarranted hold of the literature. Dangers lie in mutating aromatic residues, often key for defining the protein fold, then ascribing a direct binding role to the mutated positions without checking the structural integrity of the mutant protein. Furthermore, our analysis underscores the urgent need for experimental structural information of a complex between caveolin (or a suitable peptide) and a protein partner.

## Materials and Methods

Proteins with experimentally-demonstrated interactions with Cav-1 were collected by surveying the protein-protein interaction databases IntAct v.3.1, BioGrid^3.1^, and APID-beta [65]–[67] in conjunction with literature searches. The complete Cav-1 interactome (including proteins with multiple experimentally demonstrated interactions with Cav-1 and CBM containing proteins) compiled for this study (including Uniprot accession numbers) can be found as supporting data (Table S1). Shared motifs between caveolin-interacting proteins were sought with the SLiMFinder webserver (with or without disorder masking) using UniProt IDs as the input. Default SLiMFinder settings were altered to enable SLiMs containing up to six total wildcard positions and four consecutive wildcard positions to be included in the search criteria (disorder masking activated). In this way, CBMs corresponding to the definition of Couet *et al.* [6] would be returned if discovered with statistical significance. In this regard, it is noteworthy that the SLiMFinder webserver will identify motifs with up to five defined (i.e. non-wildcard) positions, meaning that identification of the CBM, containing just three defined positions (i.e. the functional aromatic residues), would have been possible were it a significantly enriched motif. Only returned motifs with a significance of 0.05 were considered as confident predictions [26]. The SLiMPred webserver was used to identify amino acids predicted to be part of functional SLiMs, using a threshold cutoff SLiMPred score of 0.1 [27]. Motif instances in the human proteome were identified using ps_scan [68] and sequence data obtained from UniProt [69].

Relative solvent accessibility of aromatic residues in putative CBMs was measured and changes in folding free energy (ΔΔG) resulting from alanine point mutation predicted using experimental structures where available. For other proteins, where suitable template structures were available, homology models from the SWISS-MODEL repository were used [70]. In brief, relative solvent accessible areas (RSAs) were calculated by dividing the water exposed surface area (in Å2) of a residue, measured using DSSP [71], by the total surface area of the residue. Any residue with an RSA<20% was considered buried [53]. In instances where no structure, experimental or modelled, was available, SABLE [54] was used to predict the RSA. Mutant protein stability changes were predicted by the web tool PoPMuSic v2.1 [39]. MultiProt was used for protein structure superpositions [72], the AD-ENM server for elastic network model simulations [52] and PyMOL (http://www.pymol.org) for structure visualisation. For clarity, all information regarding CBM aromatic positioning presented and discussed throughout this manuscript refers to UniProt human protein sequences, and to the canonical isoform where several are known.

## Supporting Information

Figure S1 **Comparison of all available Gi1α crystal structures in the vicinity of the CBM β-hairpin.** Each structure is drawn as a line and shown in a different colour. For comparison with Fig. 1, the aromatic residues of PDB code 1CIP are emphasised as green sticks. The PDB codes of other structures shown are 1SVS, 1AGR, 1AS0, 1AS2, 1AS3, 1BH2, 1BOF, 1CIP, 1GDD, 1GFI, 1GG2, 1GIA, 1GIL, 1GIT, 1GP2, 1KJY, 1SVK, 1Y3A, 2EBC, 2G83, 2GTP, 2HLB, 2IK8, 2OM2, 2PZ2, 2PZ3, 2XNS, 2ZJY, 2ZJZ, 3D7M, 3FFA, 3FFB and 3ONW.
(TIF)

Figure S2 **Comparison of the rat Gi1α protein (PDB code 1CIP;** [**51**] **) with structures of bovine Gsα (PDB code 1AZT; yellow), transducin (PDB code 1TAD; magenta), Arabidopsis G1α (PDB code 2XTZ; orange), and mouse G(o) subunit alpha (PDB code 3C7K; green).** The CBM aromatic residues are shown as sticks (1CIP) or as lines.
(TIF)

Figure S3 **Comparison of the rat Gi1α protein (PDB code 1CIP;** [**51**] **; motif coloured as in** **Fig. 1** **, otherwise pink) and the maximum (black) and minimum (white) projections of normal mode 8 (see text).**
(TIF)

Figure S4 **View of the context of the CBM of EGFR (A, PDB code 2J6M;** [**131**] **), insulin receptor (B, PDB code 3BU3;** [**132**] **) integrin-linked kinase (C, PDB code 3REP; Fukuda & Qin, to be published), PTEN (D, PDB code 1D5R;** [**133**] **), Slo1 (E, PDB code 3MT5;** [**134**] **), and the two CBMs of PDK1 (F and G, PDB code 1UU3;** [**135**] **).** The structures of the motifs are shown as cartoons, coloured in green, and the aromatic residues are labelled sticks. The remainder of the protein is shown as lines and surface.
(TIF)

Table S1 List of all Caveolin-1 interacting proteins.
(DOCX)

Table S2 SLiMPred scores for all CBM residues.
(DOCX)

Table S3 CBM conservation scores.
(DOCX)
