# Correction: Evaluating Caveolin Interactions: Do Proteins Interact with the Caveolin Scaffolding Domain through a Widespread Aromatic Residue-Rich Motif?

**DOI:** 10.1371/annotation/2c275a1b-2d36-4492-b36a-192bddf14f78

**Published:** 2013-02-25

**Authors:** Dominic P. Byrne, Caroline Dart, Daniel J. Rigden

There are errors in Table 4. The correct version of Table 4 can be seen here:

Part 1:

**Figure:**
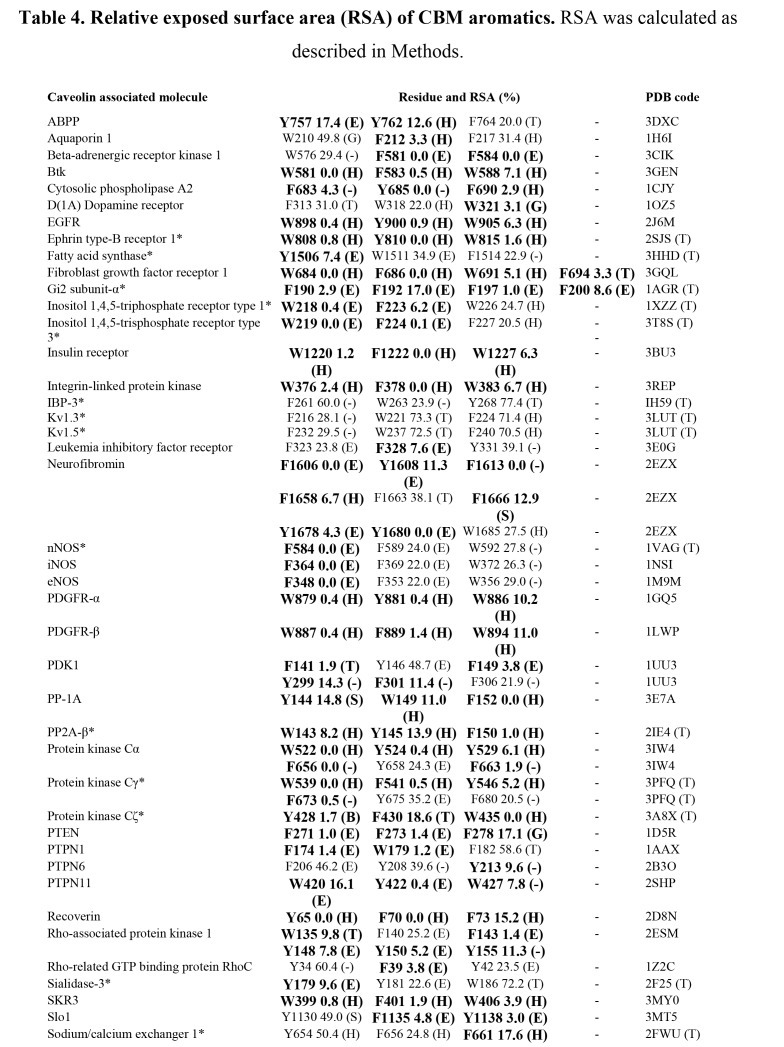

Part 2:

**Figure:**
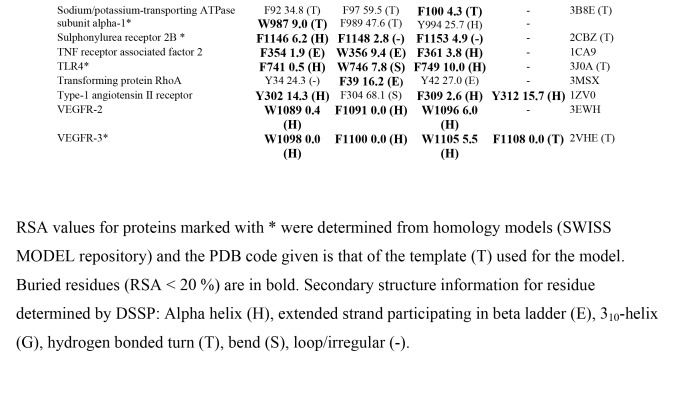

In the supplemental PDF download of this article, the Table 4 is correct, but missing the footnotes. The missing footnote information is: RSA values for proteins marked with * were determined from homology models (SWISS MODEL repository) and the PDB code given is that of the template (T) used for the model. Buried residues (RSA < 20 %) are in bold. Secondary structure information for residue determined by DSSP: Alpha helix (H), extended strand participating in beta ladder (E), 310-helix (G), hydrogen bonded turn (T), bend (S), loop/irregular (-).

