# Supplementary figures and images for: Evaluating Caveolin Interactions: Do Proteins Interact with the Caveolin Scaffolding Domain through a Widespread Aromatic Residue-Rich Motif?
Source: PLoS One. 2012 Sep 17;7(9):e44879. doi: 10.1371/journal.pone.0044879 (PMC3444507; doi:10.1371/journal.pone.0044879)

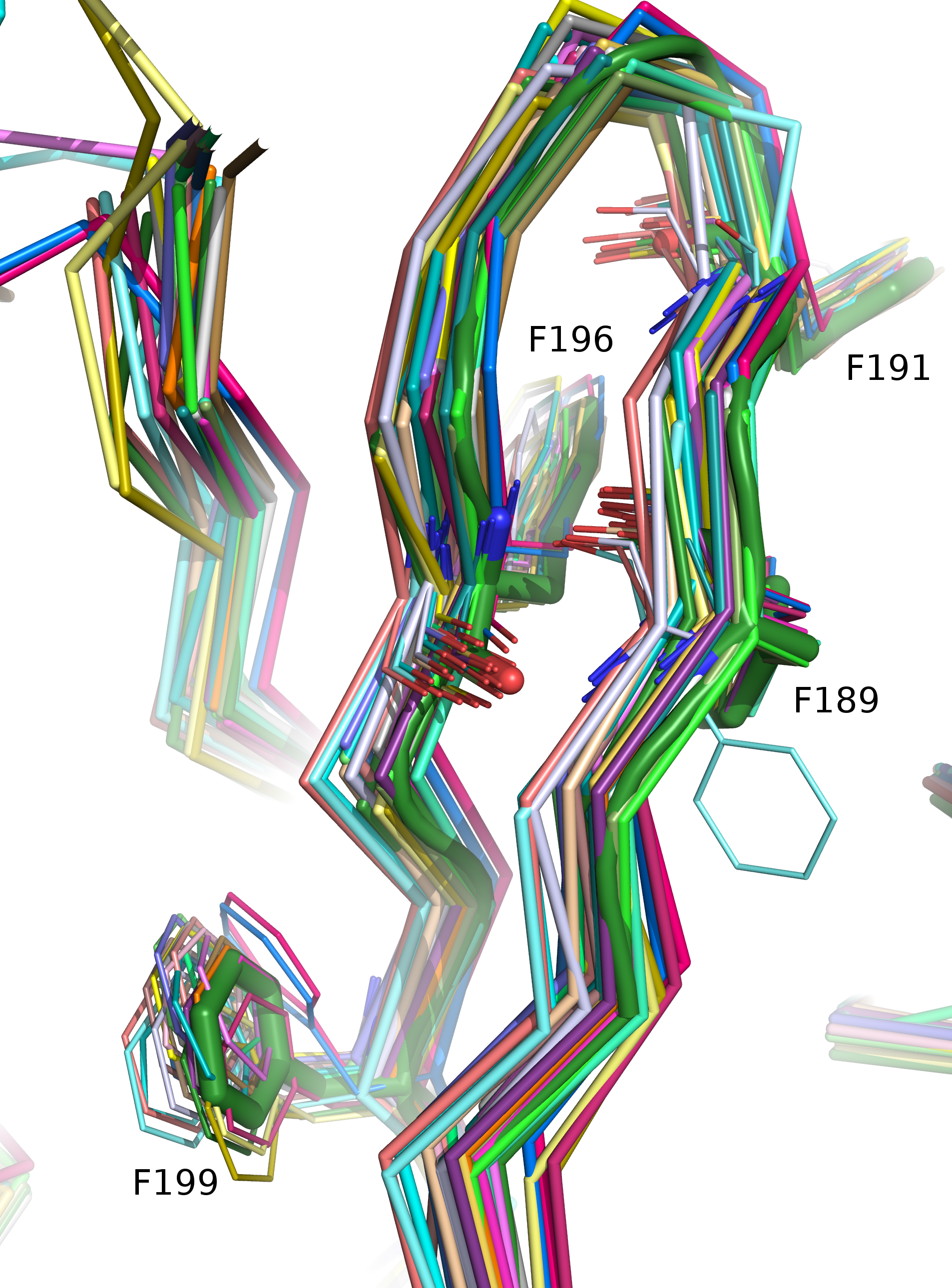

Supplement: Figure S1 — Comparison of all available Gi1α crystal structures in the vicinity of the CBM β-hairpin. Each structure is drawn as a line and shown in a different colour. For comparison with Fig. 1, the aromatic residues of PDB code 1CIP are emphasised as green sticks. The PDB codes of other structures shown are 1SVS, 1AGR, 1AS0, 1AS2, 1AS3, 1BH2, 1BOF, 1CIP, 1GDD, 1GFI, 1GG2, 1GIA, 1GIL, 1GIT, 1GP2, 1KJY, 1SVK, 1Y3A, 2EBC, 2G83, 2GTP, 2HLB, 2IK8, 2OM2, 2PZ2, 2PZ3, 2XNS, 2ZJY, 2ZJZ, 3D7M, 3FFA, 3FFB and 3ONW. (TIF) [file pone.0044879.s001.tif]

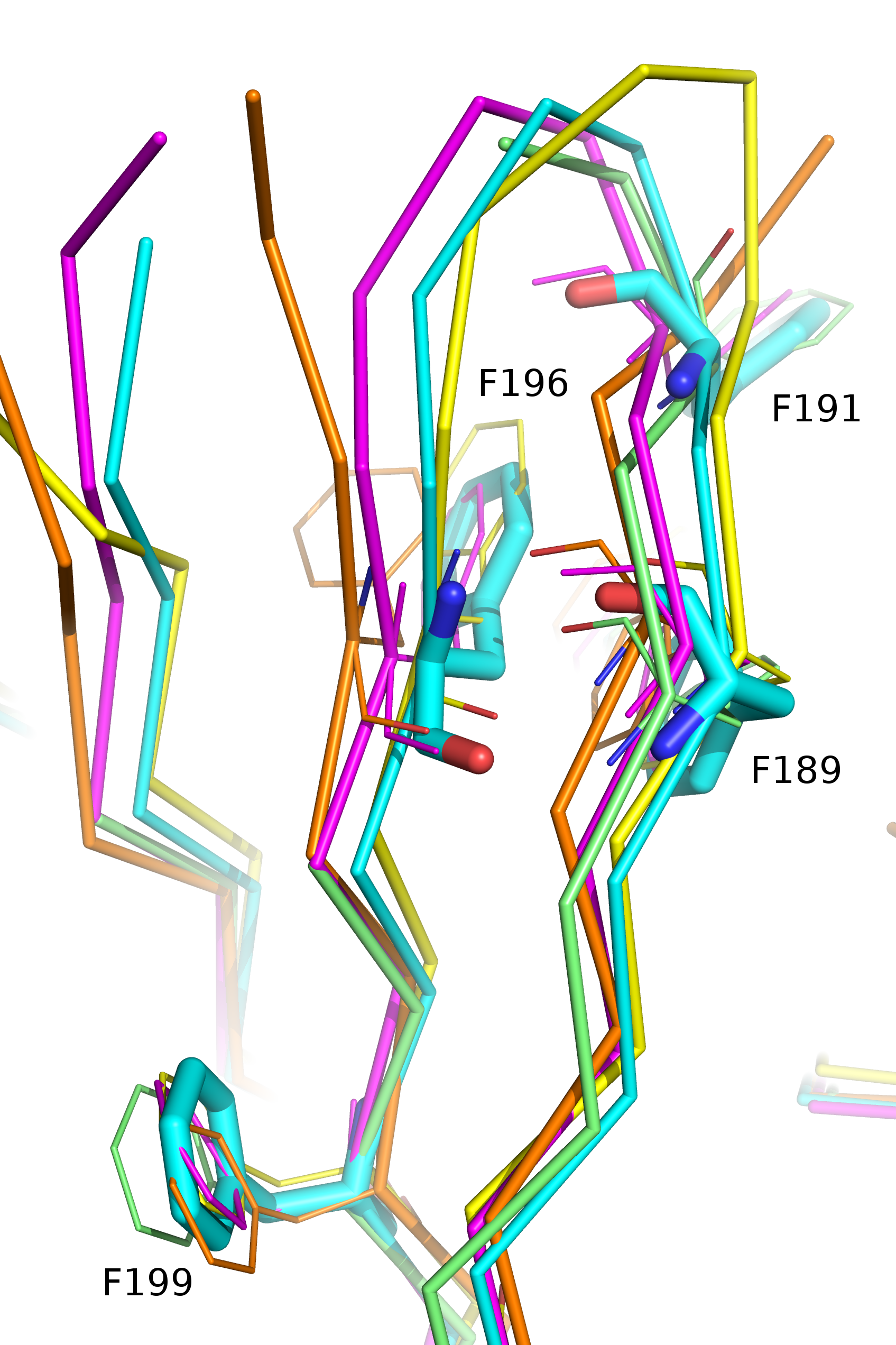

Supplement: Figure S2 — Comparison of the rat Gi1α protein (PDB code 1CIP; [51] ) with structures of bovine Gsα (PDB code 1AZT; yellow), transducin (PDB code 1TAD; magenta), Arabidopsis G1α (PDB code 2XTZ; orange), and mouse G(o) subunit alpha (PDB code 3C7K; green). The CBM aromatic residues are shown as sticks (1CIP) or as lines. (TIF) [file pone.0044879.s002.tif]

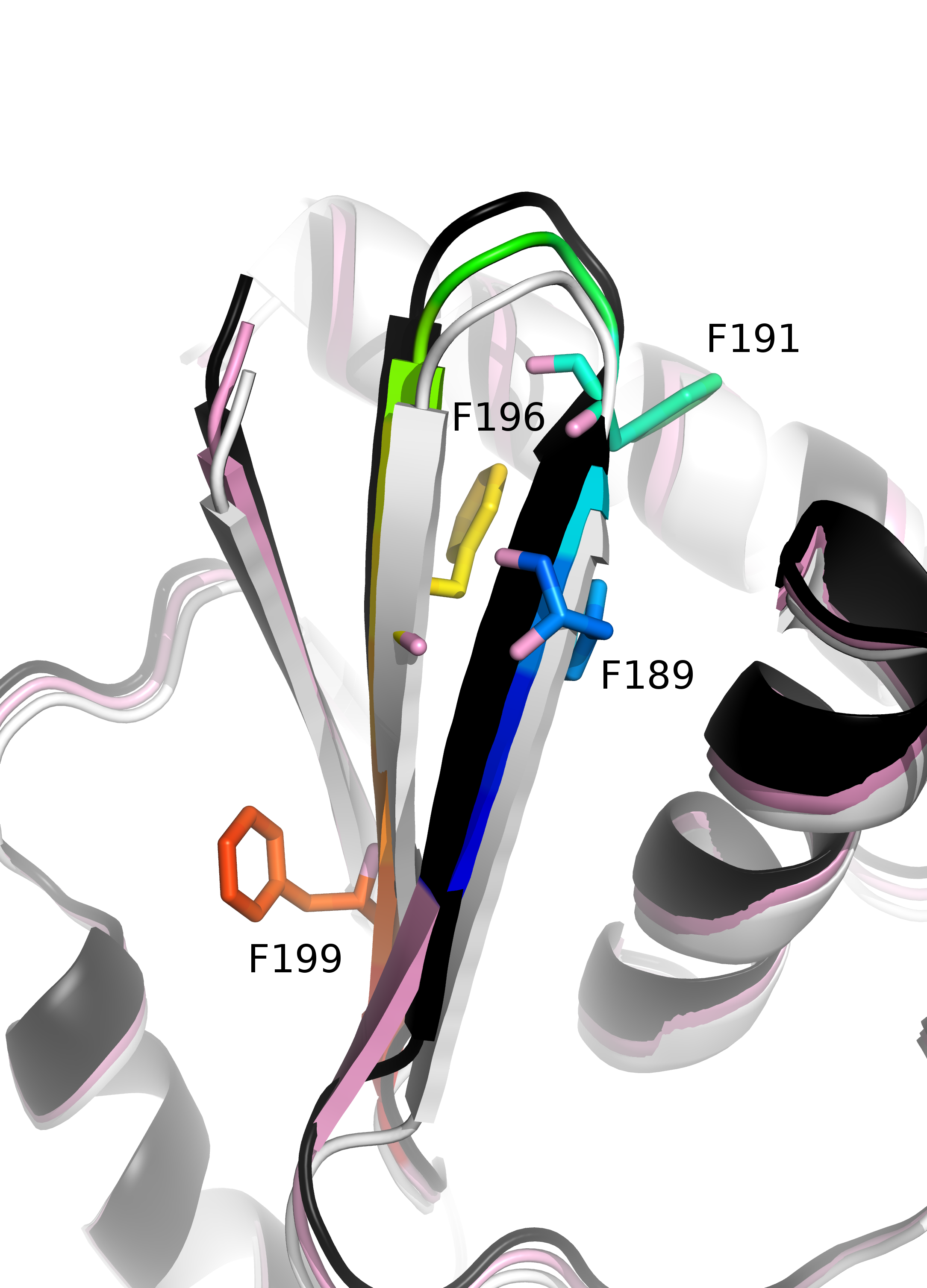

Supplement: Figure S3 — Comparison of the rat Gi1α protein (PDB code 1CIP; [51] ; motif coloured as in Fig. 1 , otherwise pink) and the maximum (black) and minimum (white) projections of normal mode 8 (see text). (TIF) [file pone.0044879.s003.tif]

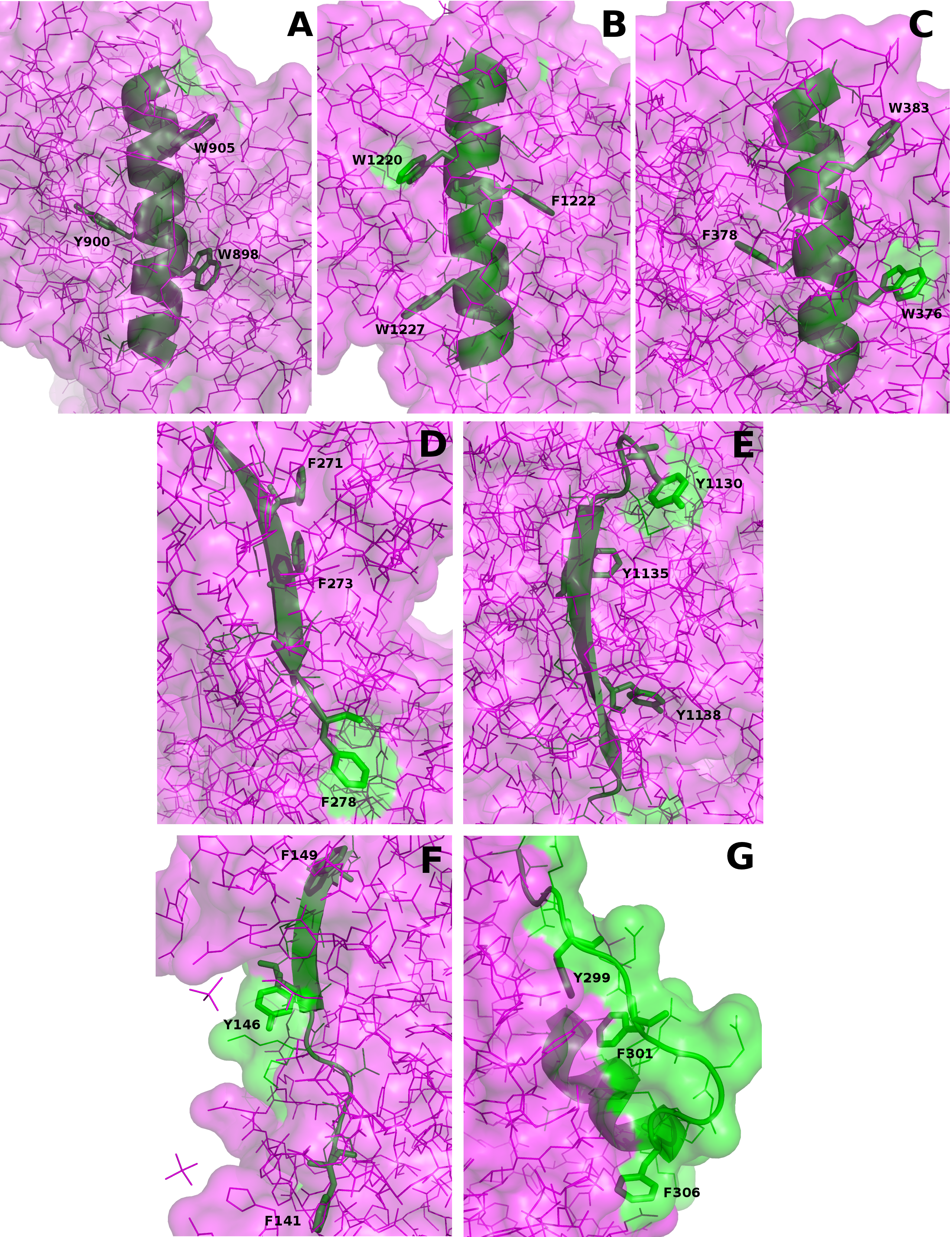

Supplement: Figure S4 — View of the context of the CBM of EGFR (A, PDB code 2J6M; [131] ), insulin receptor (B, PDB code 3BU3; [132] ) integrin-linked kinase (C, PDB code 3REP; Fukuda & Qin, to be published), PTEN (D, PDB code 1D5R; [133] ), Slo1 (E, PDB code 3MT5; [134] ), and the two CBMs of PDK1 (F and G, PDB code 1UU3; [135] ). The structures of the motifs are shown as cartoons, coloured in green, and the aromatic residues are labelled sticks. The remainder of the protein is shown as lines and surface. (TIF) [file pone.0044879.s004.tif]
