# Supplementary material for: Evaluating Caveolin Interactions: Do Proteins Interact with the Caveolin Scaffolding Domain through a Widespread Aromatic Residue-Rich Motif?
Source: PLoS One. 2012 Sep 17;7(9):e44879. doi: 10.1371/journal.pone.0044879 (PMC3444507; doi:10.1371/journal.pone.0044879)
Supplement: Table S1 — List of all Caveolin-1 interacting proteins. (DOCX) [file pone.0044879.s005.docx]

**Supporting Table S1. List of all Caveolin-1 interacting proteins**

| **Caveolin associated/CBM containing molecule** | **UniProt accession number** |
| --- | --- |
| 3-phosphoinositide-dependent protein kinase 1* | O15530 |
| 5-hydroxytryptamine receptor 2A* | P28223 |
| 72 kDa type IV collagenase* | P08253 |
| Actin, cytoplasmic 1* | P60709 |
| Adenosine receptor A1* | P30542 |
| Adenylate cyclase type 6* | O43306 |
| Amyloid beta A4 protein* | P05067 |
| Androgen receptor* | P10275 |
| Annexin A2* | P07355 |
| Apolipoprotein E* | P02649 |
| Apoptosis regulator Bcl-2* | P10415 |
| Aquaporin-1* | P29972 |
| ATP-binding cassette sub-family G member 2* | Q9UNQ0 |
| Basigin* | P35613 |
| Beta-1 adrenergic receptor* | P08588 |
| Beta-2 adrenergic receptor* | P07550 |
| Beta-adrenergic receptor kinase 1* | P25098 |
| BH3-interacting domain death agonist* | P55957 |
| Cadherin-1* | P12830 |
| Calcium-activated potassium channel subunit alpha-1* | Q12791 |
| Calcium release-activated calcium channel protein 1* | Q96D31 |
| Calpain-2 catalytic subunit* | P17655 |
| cAMP-dependent protein kinase catalytic subunit alpha* | P17612 |
| Catenin beta-1* | P35222 |
| Cell division control protein 42 homolog* | P60953 |
| cGMP-inhibited 3',5'-cyclic phosphodiesterase B* | Q13370 |
| Cytosolic phospholipase A2* | P47712 |
| D(1A) dopamine receptor* | P21728 |
| Desmoglein-2* | Q14126 |
| DNA-binding protein inhibitor ID-1* | P41135 |
| Dynamin-1* | Q05193 |
| Dynamin-2* | P50570 |
| Dystroglycan* | Q14118 |
| Endothelin A receptor* | P25101 |
| Endothelin B receptor* | P24530 |
| Epidermal growth factor receptor* | P00533 |
| Ephrin type-B receptor 1* | P54762 |
| Estrogen receptor* | P03372 |
| Estrogen receptor beta* | Q92731 |
| Extracellular calcium-sensing receptor* | P41180 |
| Fatty acid synthase* | P49327 |
| Fibroblast growth factor receptor 1* | P11362 |
| Filamin-A* | P21333 |
| Flotillin-2* | Q14254 |
| Furin* | P09958 |
| Gap junction alpha-1 protein* | P17302 |
| Gap junction alpha-3 protein* | Q9Y6H8 |
| Gap junction beta-1 protein* | P08034 |
| Gap junction beta-2 protein* | P29033 |
| Gap junction delta-2 protein* | Q9UKL4 |
| Glucagon-like peptide 1 receptor* | P43220 |
| Growth factor receptor-bound protein 7* | Q14451 |
| GTPase Hras* | P01112 |
| Guanine nucleotide-binding protein G(i) subunit alpha-2* | P04899 |
| Guanine nucleotide-binding protein G(q) subunit alpha* | P50148 |
| Heam oxygenase 1* | P09601 |
| Hepatocyte cell adhesion molecule* | Q14CZ8 |
| High affinity nerve growth factor receptor* | P04629 |
| Inositol 1,4,5-trisphosphate receptor type 1 | Q14643 |
| Inositol 1,4,5-trisphosphate receptor type 3* | Q14573 |
| Insulin-like growth factor-binding protein 3 | P17936 |
| Insulin-like growth factor-binding protein 5* | P24593 |
| Insulin receptor* | P06213 |
| Insulin receptor substrate 1* | P35568 |
| Integrin-linked protein kinase* | Q13418 |
| Interleukin-6 receptor subunit beta* | P40189 |
| Leukemia inhibitory factor receptor* | P42702 |
| Low molecular weight phosphotyrosine protein phosphatase* | P24666 |
| Major prion protein* | P04156 |
| MAL-like protein* | Q13021 |
| Matrix metalloproteinase-14* | P50281 |
| Metabotropic glutamate receptor 1* | Q13255 |
| Metalloreductase STEAP4 | Q687X5 |
| Mitogen-activated protein kinase 3* | P27361 |
| Multidrug resistance protein 1* | P08183 |
| Nitric oxide synthase, brain* | P29475 |
| Nitric oxide synthase, endothelial* | P29474 |
| Nitric oxide synthase, inducible* | P35228 |
| NEDD8* | Q15843 |
| Neurofibromin* | P21359 |
| Non-specific lipid-transfer protein* | P22307 |
| Nostrin* | Q8IVI9 |
| Peroxisome proliferator-activated receptor gamma | P37231 |
| Phosphatidylinositol 3,4,5-trisphosphate 3-phosphatase and dual-specificity* protein phosphatase PTEN | P60484 |
| Phospholipase D1* | Q13393 |
| Phospholipase D2* | O14939 |
| Platelet-derived growth factor receptor alpha* | P16234 |
| Platelet-derived growth factor receptor beta* | P09619 |
| Potassium voltage-gated channel subfamily A member 3* | P22001 |
| Potassium voltage-gated channel subfamily A member 5* | P22460 |
| Prolow-density lipoprotein receptor-related protein 1* | Q07954 |
| Prostacyclin synthase* | Q16647 |
| Prostaglandin G/H synthase 2* | P35354 |
| Proteinase-activated receptor 1* | P25116 |
| Protein kinase C alpha type* | P17252 |
| Protein kinase C gamma type* | P05129 |
| Protein kinase C zeta type* | Q05513 |
| Protein patched homolog 1* | Q13635 |
| Protein sprouty homolog 2* | O43597 |
| Proto-oncogene tyrosine-protein kinase Src* | P12931 |
| Ras-related C3 botulinum toxin substrate 1* | P63000 |
| Ras-related protein Rab-5A* | P20339 |
| Receptor-type tyrosine-protein phosphatase F* | P10586 |
| Recoverin* | P35243 |
| Rho-associated protein kinase 1* | Q13464 |
| Rho-related GTP binding protein RhoC | P08134 |
| Serine/threonine-protein kinase receptor R3* | P37023 |
| Serine/threonine-protein phosphatase 2A catalytic subunit beta isoform* | P62714 |
| Serine/threonine-protein phosphatase PP1-alpha catalytic subunit* | P62136 |
| Short transient receptor potential channel 1* | P48995 |
| Sialidase-3* | Q9UQ49 |
| Sodium/calcium exchanger 1* | P32418 |
| Sodium/potassium-transporting ATPase subunit alpha-1* | P05023 |
| Solute carrier family 22 member 8* | Q8TCC7 |
| Solute carrier family 22 member 11* | Q9NSA0 |
| Sphingosine 1-phosphate receptor 1* | P21453 |
| Stomatin-like protein 3* | Q8TAV4 |
| Striatin* | O43815 |
| Striatin-4* | Q9NRL3 |
| Sulfonylurea receptor 2B* | O60706-2 |
| Synaptosomal-associated protein 25* | P60880 |
| Toll-like receptor 4* | O00206 |
| TGF-beta receptor type-1* | P36897 |
| TNF receptor-associated factor 2* | Q12933 |
| Transforming protein RhoA* | P61586 |
| Tumor necrosis factor receptor superfamily member 5* | P25942 |
| Tumor necrosis factor receptor superfamily member 6* | P25445 |
| Tumor necrosis factor receptor superfamily member 16* | P08138 |
| Type-1 angiotensin II receptor* | P30556 |
| Tyrosine-protein phosphatase non-receptor type 1* | P18031 |
| Tyrosine-protein phosphatase non-receptor type 6* | P29350 |
| Tyrosine-protein phosphatase non-receptor type 11* | Q06124 |
| Tyrosine-protein kinase BTK* | Q06187 |
| Tyrosine-protein kinase CSK* | P41240 |
| Tyrosine-protein kinase Fyn* | P06241 |
| Tyrosine-protein kinase JAK2* | O60674 |
| Vascular endothelial growth factor receptor 2* | P35968 |
| Vascular endothelial growth factor receptor 3* | P35916 |
| Vesicle-associated membrane protein 2* | P63027 |
| Voltage-dependent anion-selective channel protein 1* | P21796 |

Proteins marked with * have multiple experimentally demonstrated interactions with Cav-1
