# Supplementary material for: Evaluating Caveolin Interactions: Do Proteins Interact with the Caveolin Scaffolding Domain through a Widespread Aromatic Residue-Rich Motif?
Source: PLoS One. 2012 Sep 17;7(9):e44879. doi: 10.1371/journal.pone.0044879 (PMC3444507; doi:10.1371/journal.pone.0044879)
Supplement: Table S2 — SLiMPred scores for all CBM residues. (DOCX) [file pone.0044879.s006.docx]

**Supporting Table S2. SLiMPred scores for all CBM residues**

| **Caveolin associated molecule** | **SLiMPred Scores** | | | | | | | | | |  |
| --- | --- | --- | --- | --- | --- | --- | --- | --- | --- | --- | --- |
| ABPP | Y757 0.07 | **E758 0.21** | **N759 0.11** | **P760 0.11** | T761 0.09 | **Y762 0.25** | **K763 0.27** | **F764 0.30** | **F764 0.29** | - | - |
| Beta-adrenergic receptor kinase 1 | W576 0.00 | Q577 0.00 | R578 0.00 | R579 0.01 | **Y580 0.10** | **F581 0.125** | Y582 0.09 | L583 0.02 | F584 0.00 | - | - |
| Btk | W581 0.00 | A582 0.00 | F583 0.05 | G584 0.00 | V585 0.00 | L586 0.00 | M587 0.00 | W588 0.00 | - | - | - |
| D(1A) Dopamine receptor | **F313 0.13** | **D314 0.29** | **V315 0.43** | **F316 0.32** | **V317 0.17** | **W318 0.13** | **F319 0.18** | **G320 0.14** | **W321 0.11** | - | - |
| EGFR | W898 0.00 | S899 0.00 | Y900 0.00 | G901 0.00 | V902 0.00 | T903 0.00 | V904 0.00 | W905 0.00 | - | - | - |
| Ephrin type-B receptor 1 | W808 0.00 | S809 0.00 | Y810 0.00 | G811 0.00 | I812 0.00 | V813 0.00 | M814 0.00 | W815 0.00 | - | - | - |
| Gi2 subunit-α | F190 0.00 | T191 0.00 | F192 0.00 | K193 0.00 | D194 0.00 | L195 0.00 | H196 0.00 | F197 0.00 | - | - | - |
| Glucagon-like peptide 1 receptor | Y250 0.09 | L251 0.07 | Y252 0.09 | T253 0.04 | **L254 0.20** | **L255 0.21** | **A256 0.13** | **F257 0.10** | - | - | - |
| Insulin receptor | W1220 0.00 | S1221 0.00 | F1222 0.00 | G1223 0.00 | V1224 0.03 | V1225 0.00 | L1226 0.00 | W1227 0.00 | - | - | - |
| Integrin-linked protein kinase | W376 0.00 | S377 0.00 | F378 0.00 | A379 0.01 | V380 0.01 | L381 0.06 | L382 0.06 | W383 0.06 | - | - | - |
| Metabolic glutamate receptor 1 | **F609 0.46** | **V610 0.18** | **T611 0.12** | L612 0.08 | I613 0.06 | F614 0.05 | V615 0.08 | L616 0.08 | **Y617 0.12** | - | - |
|  | F781 0.08 | N782 0.09 | E783 0.00 | A784 0.00 | **K785 0.26** | **Y786 0.15** | **I787 0.14** | **A788 0.17** | **Y789 0.11** | - | - |
| Multidrug resistance protein 1 | **F37 0.43** | **S38 0.28** | **M39 0.11** | **F40 0.13** | **R41 0.17** | **Y42 0.33** | **S43 0.21** | N44 0.06 | **W45 0.17** | - | - |
| nNOS | F584 0.00 | S585 0.00 | A586 0.00 | C587 0.00 | P588 0.00 | F589 0.00 | S590 0.00 | G591 0.00 | W592 0.00 | - | - |
| eNOS | F348 0.00 | P349 0.00 | A350 0.03 | A351 0.02 | P352 0.00 | F353 0.00 | S354 0.00 | G355 0.00 | W356 0.00 | - | - |
| PDK1 | F141 0.00 | F142 0.00 | V143 0.00 | K144 0.00 | L145 0.00 | Y146 0.00 | F147 0.00 | T148 0.00 | F149 0.00 | - | - |
|  | Y299 0.08 | **D300 0.24** | F301 0.09 | **P302 0.14** | E303 0.00 | K304 0.03 | F305 0.02 | F306 0.01 | - | - | - |
| Ptc | Y788 0.00 | D789 0.01 | **F790 0.46** | **I791 0.46** | **A792 0.43** | **A793 0.28** | **Q294 0.35** | **F795 0.56** | **K796 0.56** | **Y797 0.61** | **F798 0.51** |
| PTEN | F271 0.00 | H272 0.00 | F273 0.00 | W274 0.00 | V275 0.00 | N276 0.00 | T277 0.00 | F278 0.00 | - | - | - |
| Sialidase-3 | Y179 0.00 | T180 0.00 | Y181 0.00 | Y182 0.00 | I183 0.00 | P184 0.05 | S185 0.00 | W186 0.00 | - | - | - |
| SKR3 | W399 0.00 | A400 0.00 | F401 0.00 | G402 0.00 | L403 0.05 | V404 0.09 | L405 0.09 | W406 0.04 | - | - | - |
| Slo1 | Y1130 0.05 | N1131 0.09 | **M1132 0.17** | **L1133 0.14** | **C1134 0.19** | **F1135 0.18** | G1136 0.09 | I1139 0.03 | Y1138 0.02 | - | - |
| Sodium/potassium-transporting ATPase subunit alpha-1 | **F92 0.10** | C93 0.07 | R94 0.06 | **Q95 0.18** | **L96 0.15** | F97 0.05 | G98 0.01 | **G99 0.1** | F100 0.08 | - | - |
|  | **W987 0.39** | **W988 0.43** | **F989 0.55** | **C990 0.38** | **A991 0.28** | **F992 0.41** | **P993 0.37** | **Y994 0.40** | - | - | - |
| Striatin | **F55 0.42** | **L56 0.12** | Q57 0.08 | H58 0.08 | **E59 0.16** | **W60 0.15** | **A61 0.12** | **R62 0.25** | **F63 0.25** | - | - |
| TLR4 | F741 0.00 | I742 0.00 | Q743 0.00 | S744 0.00 | R745 0.00 | **W746 0.18** | C747 0.06 | I748 0.06 | F749 0.09 | - | - |
| TrpC1 | **F781 0.12** | R82 0.07 | **T83 0.15** | **S84 0.32** | **K85 0.17** | **Y86 0.53** | **A87 0.39** | **M88 0.28** | **F789 0.33** | - | - |
| Type-1 angiotensin II receptor | **Y302 0.11** | G303 0.00 | F304 0.00 | L305 0.00 | G306 0.05 | K307 0.00 | K308 0.00 | F309 0.05 | K310 0.06 | **R311 0.18** | **Y312 0.12** |

SLiMPred webserver was run on proteins with experimental evidence suggesting the CBM facilitates binding to Cav-1. Predicted SLiM residues (SLiMPred score > 0.1) are in bold.
