# Supplementary material for: Evaluating Caveolin Interactions: Do Proteins Interact with the Caveolin Scaffolding Domain through a Widespread Aromatic Residue-Rich Motif?
Source: PLoS One. 2012 Sep 17;7(9):e44879. doi: 10.1371/journal.pone.0044879 (PMC3444507; doi:10.1371/journal.pone.0044879)
Supplement: Table S3 — CBM conservation scores. (DOCX) [file pone.0044879.s007.docx]

**Supporting Table S3. CBM conservation scores derived from the ELM Conservation Scorer**

| **Caveolin associated molecule** | **CBM sequences and location (aromatic positions emboldened)** | **Conservation score** |
| --- | --- | --- |
| ABPP | 757-**Y**ENPT**Y**KF**F**-764 | 1.00 |
| Adenosine receptor A1 | 288-**Y**A**F**RIQK**F**-295 | 1.00 |
| Aquaporin 1 | 210-**W**I**F**WVGP**F**-217 | 0.42 |
| Beta-adrenergic receptor kinase 1 | 576-**W**QRRY**F**YQ**F**-584 | 0.44 |
| Btk | 581-**W**A**F**GVLM**W**-588 | 1.00 |
| cGMP-inhibited 3',5'-cyclic phosphodiesterase B | 47-**F**F**F**HLCR**F**-54 | 1.00 |
|  | 330-**W**D**W**DLKQ**W**-337 | 1.00 |
| Cytosolic phospholipase A2 | 683-**F**Q**Y**PNQA**F**-690 | 1.00 |
| D(1A) Dopamine receptor | 313-**F**DVFV**W**FG**W**-321 | 1.00 |
| EGFR | 898-**W**S**Y**GVTV**W**-905 | 1.00 |
| Ephrin type-B receptor 1 | 808-**W**S**Y**GVTV**W**-815 | 1.00 |
| Fatty acid synthase | 1506-**Y**RDGA**W**GA**F**-1514 | 0.98 |
| Fibroblast growth factor receptor 1 | 684-**W**S**F**GVLL**W**EI**F**-694 | 1.00 |
| Gi2 subunit-α | 190-**F**T**F**KDLH**F**KM**F**-200 | 0.74 |
| Glucagon-like peptide 1 receptor | 247-EGV**Y**L**Y**TLLA**F**-257 | 0.48 |
| Inositol 1,4,5-triphosphate receptor type 1 | 218-**W**KIVL**F**MK**W**-226 | 1.00 |
|  | 2461-**Y**L**F**SIVG**Y**-2468 | 1.00 |
| Inositol 1,4,5-trisphosphate receptor type 3 | 219-**W**KINL**F**MQ**F**-227 | 1.00 |
|  | 729-**Y**R**Y**QLKL**F**-736 | 1.00 |
|  | 2381-**Y**L**F**SIVG**F**L**F**LKDD**F**-2395 | 1.00 |
| Insulin receptor | 1220-**W**S**F**GVVL**W**-1227 | 1.00 |
| Integrin-linked protein kinase | 376-**W**S**F**AVLL**W**-383 | 1.00 |
| IBP-3 | 261-**F**C**W**CVDK**Y**-268 | 0.24 |
| Interleukin-6 receptor subunit beta | 606-**F**T**F**TTPK**F**-613 | 1.00 |
| Kv1.3 | 216-**F**QRQV**W**LL**F**-224 | 1.00 |
| Kv1.5 | 232-**F**QRQV**W**LI**F**-240 | 1.00 |
| Leukemia inhibitory factor receptor | 323-**F**GTVV**F**AG**Y**-331 | 0.06 |
| MAL-like protein | 143-**Y**ILHA**F**SI**Y**-151 | 0.37 |
|  | 23-**F**LTIP**F**AF**F**-31 | 0.02 |
| Metabolic glutamate receptor 1 | 609-**F**VTLI**F**VL**Y**-617 | 1.00 |
|  | 781-**F**NEAK**Y**IA**F**-789 | 1.00 |
| Metalloreductase STEAP4 | 192-**Y**PLQL**F**PM**W**-200 | 0.26 |
|  | 271-**Y**RGTK**Y**RR**F**-279 | 1.00 |
| Multidrug resistance protein 1 | 37-**F**SM**F**RYSN**W**-45 | 0.54 |
| Neurofibromin | 1606-**F**Y**Y**VARR**F**-1613 | 1.00 |
|  | 1658-**F**LSKW**F**VV**F**-1666 | 1.00 |
|  | 1678-**Y**I**Y**NCNS**W**-1685 | 1.00 |
|  | 2102-**Y**L**F**HVVF**F**-2109 | 0.75 |
| nNOS | 584-**F**SACP**F**SG**W**-592 | 1.00 |
| iNOS | 364-**F**PGCP**F**NG**W**-372 | 1.00 |
| eNOS | 348-**F**PAAP**F**SG**W**-356 | 1.00 |
| PDGFR-α | 879-**W**S**Y**GILL**W**-886 | 1.00 |
| PDGFR-β | 887-**W**S**F**GILL**W**-894 | 1.00 |
| PDK1 | 141-**F**FVKL**Y**FT**F**-149 | 1.00 |
|  | 299-**Y**D**F**PEKF**F**-306 | 0.08 |
| PP-1A | 144-**Y**NIKL**W**KT**F**-152 | 1.00 |
| PP2A-β | 143-**W**K**Y**FTDL**F**-150 | 0.46 |
| Protein kinase Cα | 522-**W**A**Y**GVLL**Y**-529 | 0.95 |
|  | 656-**F**S**Y**VNPQ**F**-663 | 1.00 |
| Protein kinase Cγ | 539-**W**S**F**GVLL**Y**-546 | 1.00 |
|  | 673-**F**T**Y**VNPD**F**-680 | 1.00 |
| Protein kinase Cζ | 428-**Y**G**F**SVDW**W**-435 | 0.98 |
| Ptc | 788-**Y**D**F**IAAQ**F**KY**F**-798 | 0.18 |
| PTEN | 174-**F**HYTT**W**PD**F**-182 | 1.00 |
| PTPN1 | 271-**F**H**F**WVNT**F**-278 | 1.00 |
| PTPN6 | 206-**F**V**Y**LRQP**Y**-213 | 0.10 |
| PTPN11 | 420-**W**Q**Y**HFRT**W**-427 | 1.00 |
| Recoverin | 65-**Y**AQHY**F**RS**F**-73 | 1.00 |
| Rho-associated protein kinase 1 | 135-**W**VVQL**F**CAF-143 | 0.14 |
|  | 148-**Y**L**Y**MVME**Y**-155 | 1.00 |
| Rho-related GTP binding protein RhoC | 34-**Y**VPTV**F**EN**Y**-42 | 0.92 |
| Sialidase-3 | 179-**Y**T**Y**YIPS**W**-186 | 0.06 |
| SKR3 | 399-**W**A**F**GLVL**W**-406 | 0.92 |
| Slo1 | 1130-**Y**NMLC**F**GI**Y**-1138 | 1.00 |
| Sodium/calcium exchanger 1 | 259-**Y**K**Y**VYKR**Y**-266 | 1.00 |
|  | 654-**Y**L**F**GQPV**F**-661 | 0.09 |
| Sodium/potassium-transporting ATPase subunit alpha-1 | 92-**F**CRQL**F**GG**F**-100 | 0.93 |
|  | 987-**W**W**F**CAFP**Y**-994 | 0.72 |
| Solute carrier family 22 member 11 | 158-**F**I**W**GLLS**Y**-165 | 0.00 |
| Solute carrier family 22 member 8 | 216-**Y**C**Y**TFGQ**F**-223 | 0.18 |
| Striatin | 55-**F**LQHE**W**AR**F**-63 | 1.00 |
| Striatin-4 | 71-**F**IQHE**W**AR**F**-79 | 1.00 |
| Sulphonylurea receptor 2B | 138-**F**L**Y**WVMA**F**-145 | 0.04 |
|  | 1146-**F**Y**F**IQKY**F**-1153 | 0.96 |
| TLR4 | 741-**F**IQSR**W**CI**F**-749 | 1.00 |
| TNF receptor associated factor 2 | 354-**F**I**W**KISD**F**-361 | 1.00 |
| Transforming protein RhoA | 34-**Y**VPTV**F**EN**Y**-42 | 0.88 |
| TrpC1 | 781-**F**RTSK**Y**AM**F**-789 | 0.07 |
| Type-1 angiotensin II receptor | 302-**Y**G**F**LGKK**F**KR**Y**-312 | 0.24 |
| VEGFR-2 | 1089-**W**S**F**GVKK**W**EI**F**-1099 | 1.00 |
| VEGFR-3 | 1098-**W**S**F**GVLL**W**EI**F**-1108 | 1.00 |
